# Functional networks inference from rule-based machine learning models

**DOI:** 10.1186/s13040-016-0106-4

**Published:** 2016-09-05

**Authors:** Nicola Lazzarini, Paweł, Widera, Stuart Williamson, Rakesh Heer, Natalio Krasnogor, Jaume Bacardit

**Affiliations:** 1 Interdisciplinary Computing and Complex BioSystems (ICOS) research group, School of Computing Science, Newcastle University, Newcastle upon Tyne, UK; 2 Clinical and Experimental Pharmacology Group, Cancer Research UK Manchester Institute, University of Manchester, Manchester, UK; 3 Northern Institute for Cancer Research, Medical School, Newcastle University, Newcastle upon Tyne, UK

**Keywords:** Machine learning, Biological knowledge extraction, Network inference, Functional networks

## Abstract

**Background:**

Functional networks play an important role in the analysis of biological processes and systems. The inference of these networks from high-throughput (-omics) data is an area of intense research. So far, the similarity-based inference paradigm (e.g. gene co-expression) has been the most popular approach. It assumes a functional relationship between genes which are expressed at similar levels across different samples. An alternative to this paradigm is the inference of relationships from the structure of machine learning models. These models are able to capture complex relationships between variables, that often are different/complementary to the similarity-based methods.

**Results:**

We propose a protocol to infer functional networks from machine learning models, called FuNeL. It assumes, that genes used together within a rule-based machine learning model to classify the samples, might also be functionally related at a biological level. The protocol is first tested on synthetic datasets and then evaluated on a test suite of 8 real-world datasets related to human cancer. The networks inferred from the real-world data are compared against gene co-expression networks of equal size, generated with 3 different methods. The comparison is performed from two different points of view. We analyse the enriched biological terms in the set of network nodes and the relationships between known disease-associated genes in a context of the network topology. The comparison confirms both the biological relevance and the complementary character of the knowledge captured by the FuNeL networks in relation to similarity-based methods and demonstrates its potential to identify known disease associations as core elements of the network. Finally, using a prostate cancer dataset as a case study, we confirm that the biological knowledge captured by our method is relevant to the disease and consistent with the specialised literature and with an independent dataset not used in the inference process.

**Availability:**

The implementation of our network inference protocol is available at: http://ico2s.org/software/funel.html

**Electronic supplementary material:**

The online version of this article (doi:10.1186/s13040-016-0106-4) contains supplementary material, which is available to authorized users.

## Background

The inference of biological networks is a highly relevant and challenging task in systems biology and integrative bioinformatics. Biological networks are graphs in which nodes represent genes or proteins, and a connection between them indicates some kind of biological relationship, e.g. regulatory or functional. The network inference is, in an essence, an attempt to reverse engineer the biological relationships from the high-throughput biological data [1].

Most biological network inference methods focus on the definition of gene regulatory networks, in which edges represent direct regulatory interactions between genes [2–4]. Far less effort has been put into the design of methods to build functional networks in which a connection indicates a functional relationship, e.g. membership in the same pathway or protein complex. One of the typical uses of these networks is the identification of functional modules (subset of genes with multiple internal connections and a few connections with genes outside the module that describe, explain or predict a biological process or phenotype).

One of the earliest (but still widely used) approach to infer functional networks is the “guilt-by-association” principle [5]. That is, if two genes show *similar* expression profiles, it is assumed they are also functionally related (via a direct or indirect interaction). Initially, this paradigm was applied to infer networks from transcriptomics data, and this is why in most of the literature it is known as the *co-expression* network inference principle. Nevertheless, it is abstract enough to be applied to all kinds of biological data. It has been demonstrated that co-expression networks are able to effectively identify pathways and candidate biomarkers [6] or reveal gene modules representing a biological process perturbed in a disease [7], just to name a few examples, and the similarity-based approach remains the dominant method of functional network inference today, with many recent examples: [8–12].

**Fig. 1:**
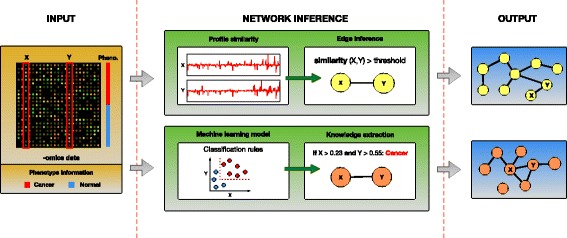
Two approaches to functional network inference: one based on the expression profile similarity and the other based on the extraction of knowledge from machine learning models. The similarity-based methods construct a new network edge *X*⇔*Y*, when the similarity between the expressions of genes *X* and *Y* across the samples is above a threshold. Methods based on machine learning, first build a predictive model, in this example a rule-based model, using the samples phenotype (class labels) information and then construct a network edge *X*⇔*Y*, when genes *X* and *Y* are used together within that model to classify the samples. As these two approaches lead to different functional networks, it is possible that they capture complementary knowledge

A different approach that is recently gaining popularity, is the use of machine learning techniques to infer biological networks. Due to the wide range of knowledge representations used within machine learning methods (e.g. classification rules, decision trees, artificial neural networks, SVM kernels, etc.), they can discover more complex and diverse relationships, and overcome the limitations of the similarity-based methods. This is possible because within machine learning models the attributes are associated not because they are similar (e.g. have similar expression profiles), but because together they detect strong patterns. In addition, if learning is supervised, it can take advantage of the additional phenotype information (class labels of the samples, e.g. case and control) available with the data. Therefore, by mining the complex machine learning models, it should be possible to uncover new and different (biological) knowledge, that is likely to escape the traditional approaches. Figure 1 illustrates these differences between the two approaches (similarity-based methods vs. knowledge extraction from the machine learning models).

Alternative strategies exist to infer networks using machine learning. One approach is to train machine learning models that directly predict network edges [13], but this process requires an experimentally verified “ground truth” of known interactions and suitable controls. A different approach, which is the focus of this work, is to generate machine learning models from the biological data and then *mine* the structure of the models to infer networks. Several types of machine learning have been successfully applied to this task: unsupervised learning in the form of association rules [14], supervised learning using regression (model trees [15]) or classification (random forest [16]).

The specific focus of this paper is the network inference from rule-based machine learning models, these have been successfully applied before to extract knowledge from genetic data [17] and identify disease risk factors in a bladder cancer study [18]. The methods presented in these works share some pipeline components with our current work, such as the permutation test and a 2-phase learning strategy. In our previous works we applied rule-based machine learning to transcriptomics [19, 20], proteomics [21], lipidomics [22] and protein structure data [23]. We formulated a paradigm called *co-prediction* (in opposition to the classic co-expression) in which the prediction rules of a classification algorithm, in our case BioHEL [24], are used to identify relationships between genes.

**Fig. 2:**
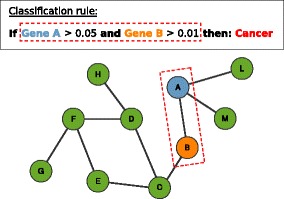
Co-prediction paradigm. Association between the genes is inferred from their co-occurrence in classification rules

Co-prediction is based on the assumption that attributes (e.g. genes) within the same classification rules, due to their co-operation in predicting the sample class, have an increased likelihood of being functionally related to the biological process in question (Fig. 2). Differently than co-expression, the co-prediction approach exploits the phenotype information of the data (class labels) to detect functional relations.

However, from a methodological perspective, many questions remained unanswered. Can the co-prediction approach identify known genetic relationships? How can we quantify the biological significance of the co-prediction networks? What is the impact of data pre-processing on the generated networks? Is this methodology able to capture knowledge that escapes other methods? Are the discovered functional relationships meaningful in the human disease context?

To address these questions, we propose in this article a new network inference protocol, called FuNeL (Functional Network Learning). FuNeL substantially extends our previous work [19] by incorporating: (1) statistical filtering of inferred functional relationships via permutation tests, (2) a multi-stage network generation to maximise the knowledge extraction, and (3) a configurable feature selection stage to control the size of the generated networks.

We first tested FuNeL’s ability to correctly identify functional relationships using a set of synthetic datasets. Then, we evaluated FuNeL on 8 real-world transcriptomics datasets related to different types of cancer. For each dataset we tested 4 different configurations of the protocol and compared the inferred networks to co-expression networks of equivalent size. In order to have an extensive evaluation of our approach, we employed 3 different methods to generate co-expression networks. We systematically looked at the differences between co-prediction and co-expression networks from two points of view: (1) the enriched biological terms and (2) the relationships between the genes known to be associated with a particular type of cancer. Finally, we used a prostate cancer dataset as a case study and performed a more detailed biological analysis of the enriched terms and the disease related genes. We looked at the largest hubs and the most central nodes in the prostate cancer co-prediction networks and studied their involvement in the disease. We found literature support for the association between these topologically important genes and prostate cancer, and we further confirmed it with an independent transcriptomics dataset (not used as a source in the inference process). Overall, we found that the FuNeL inferred networks: (1) capture relevant biological knowledge that is complementary to the knowledge captured by different co-expression networks, and (2) more adequately represent the relationships between genes associated with the disease targeted by each dataset.

## Materials and methods

In this section we describe the proposed network inference protocol, the datasets from which we inferred the networks and the experimental design we used to evaluate it.

### The functional network inference protocol

**Fig. 3:**
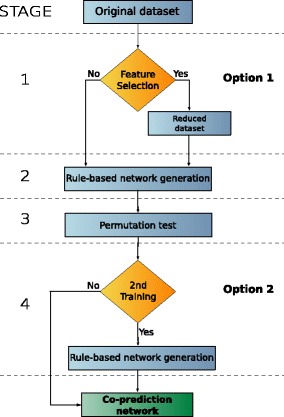
Stages of the functional network inference protocol

The stages of the co-prediction inference protocol are illustrated in Fig. 3. Two of these stages are optional (1 and 4), they lead to a total of 4 different protocol configurations. If the first optional stage (feature selection) is performed, the original dataset is reduced to the most relevant attributes. In the second stage a rule-based machine learning is used to infer a network. This network is statistically refined in Stage 3, in which a permutation test is used to filter out non-significant nodes. The final stage, in which the network generation is repeated for the second time, is again optional. A complete time complexity analysis of the FuNeL protocol is available in Section 2 of the Additional file 1: Supplementary Material.

**Feature selection (stage 1)**

When datasets contain a large number of attributes, some might be irrelevant to the prediction target and discarding them helps the classification algorithm to focus its learning effort on the attributes that matters. Therefore, the feature selection is the first stage of the inference process. To pick the relevant attributes we used the support vector machine recursive feature elimination (SVM-RFE) [25]. We opted for the SVM algorithm with a linear kernel as our preliminary studies suggested that it can eliminate as much as 90 % of the original dataset attributes, without losing much of the classification accuracy (see Section 1 in Additional file 1: Supplementary Material).

**Rule-based network inference (stage 2)**

To infer the rule-based classification models we used BioHEL [24]. It generates sets of classification rules using a genetic algorithm and is able to work with large datasets. Due to the stochastic nature of BioHEL’s learning process, each of its runs generates a different rule set. We leverage this fact by creating a large number of alternative hypotheses of functional relationships via multiple runs of the algorithm. For each dataset we run BioHEL 10,000 times and infer the network from the *consensus* of all the generated rule sets. To do that, we use all the pairs of attributes that appear together in the same classification rule as the network edges (co-prediction paradigm). Then, we score each network node (attribute) by counting how many times it has been used in the rules (node score).

**Permutation test (stage 3)**

Given a list of edges (attribute-attribute associations) extracted from the rule sets, we try to filter out the non-significant nodes. To determine the node significance, we follow a statistical analysis procedure based on a permutation test, similar to the one described in [17]. We generate 100 permutated datasets by randomly shuffling the class labels. Next, we infer the co-prediction networks (as in Stage 2) from these permutated datasets. Then, for each node, we calculate a distribution of scores across the 100 networks generated from the permutated datasets. Using a one-tailed permutation test, we assign to each node a *p*-value, to estimate how likely it is to draw its score from the calculated distribution. With this process we make sure that the nodes with high scores are really tied to the classes present in the data, and that the network truly represents functional relationships. To decide if a node is statistically significant we use a typical *α*=0.05 threshold.

After preliminary experiments we realised, that using significant nodes alone leads to small and dense networks. To counter that, we relaxed the node pruning to also keep all direct neighbours of the significant nodes.

**Network construction (stage 4)**

There are two ways to interpret the result of the statistical test (option 2 in Fig. 3). The first approach is to use the significant nodes as a filter for the inferred relationships (edges) and remove all the edges between two non-significant nodes. The second approach is to use the permutation test as a further feature selection and build a new rule-based machine learning model using only the significant nodes. This second run of the learning algorithm is then focused only on the statistically important genes and creates the final network.

**Protocol configurations**

As a result of two independent optional stages in the FuNeL protocol, there are 4 different configurations that it can run with (see Table 1). We decided to test them all and infer four networks from each dataset, one per configuration.

**Table 1:** Protocol configurations used in the experiments

Configuration	Description
*C*_1_	Reduced dataset + 1 stage of network generation
*C*_2_	Original dataset + 1 stage of network generation
*C*_3_	Reduced dataset + 2 stages of network generation
*C*_4_	Original dataset + 2 stages of network generation

### Datasets

#### Synthetic datasets

To verify if FuNeL is able to correctly identify functional relationships we tested it on a set of synthetic datasets. Although there are several generators that model expression data with genetic relationships, such as GNW used in several DREAM challenges [26], they generate unlabeled samples (without phenotype information, e.g. case vs. control) and the class labels are necessary to perform the supervised learning at the core of FuNeL.

For that reason, we decided to use GAMETES instance generator [27], designed to create genetic datasets with multi-locus disease associations, where no fewer than *n* loci can predict a phenotype (disease status). GAMETES generates genotype data (rather than gene expression data) based on models with specific genetic constraints, e.g. different heritabilities or frequencies of the SNPs.

To generate the synthetic datasets, we used a set of 2-locus configurations similar to what was employed in a recent work of Li et al. [28] to evaluate permuted random forest networks of gene interactions. Specifically, the genetic models varied in terms of heritability (0.001–0.4) and number of attributes (5–25), with fixed allele frequency of 0.2 and 2000 samples per dataset. For each configuration, we selected from 100 000 random models, two models with extreme value of the ease of detection metric (EDM) (the least and the most difficult). Finally, for each selected model we generated 50 datasets, obtaining 4000 datasets in total.

#### Real-world datasets

We used 8 publicly available human cancer microarray datasets (see Table 2). These datasets represent a broad range of characteristics in terms of biological information (different types of cancers), number of samples (patients) and number of attributes (genes). For each dataset the attributes were defined by the probes used in the microarray experiment. Generally, a gene can be represented by more than one probe and extra post-processing step is needed to merge the information and generate networks where nodes truly represent genes. We used MADGene [29] to map the Affymetrix probe IDs into HUGO gene IDs, then for all probes mapped to the same gene, we merged the probes and their connections. If a probe was unmapped it was removed from the network.

**Table 2:** Description of the source datasets used to infer networks

Name	Attributes	Samples	Class labels
Dlbcl [63]	2647	77	Dlbcl; Follicular lymphoma
CNS [64]	7129	60	Survivor; Failures
Leukemia [65]	7129	72	AML; ALL
Lung-Michigan [66]	7129	96	Tumor; Normal
Lung-Harvard [67]	12534	181	Mesothelioma; ADCA
Prostate [41]	12600	102	Tumor; Normal
AML [68]	12625	54	Remission; Relapse
Colon-Breast [69]	22283	52	Colon cancer; Breast cancer

While in this instance we focused on transcriptomics datasets only, the FuNeL protocol is general and can be applied to other types of biological data too (proteomics, lipidomics, etc.).

### Co-expression networks

In this paper we are comparing our FuNeL networks against co-expression networks. The co-expression paradigm identifies similarity of gene expression pattern under different experimental conditions. Co-expression edges are an abstraction of functional relationships between genes and do not represent physical binding as in protein interaction or gene regulatory networks. Two genes are considered to be functionally related (co-expressed), if their transcript levels are similar across a set of samples.

In here we employed three well known methods to infer co-expression networks, each one uses a different metric to assess gene expressions similarity: Pearson correlation coefficient, ARACNE [2] and MIC [30]. In the following subsections we briefly present those methods, for more details check the cited original papers.

#### Pearson correlation coefficient

Pearson’s correlation coefficient (PCC) is a well known measure of linear dependence between two variables. Applied to gene expression profiles, it measures the similarity in the direction of gene response across samples. Its main disadvantages are the lack of distributional robustness (it assumes data normality) and the sensitivity to outliers. We generated the PCC-based co-expression networks using the *SciPy* Python library [31].

#### ARACNE: algorithm for the reconstruction of gene regulatory networks

The ARACNE method [2] measures the dependence between two gene expression profiles using mutual information. Mutual information *I*(*X*;*Y*) estimates entropy to quantify the amount of information that *Y* contains about *X* (measured in bits). In contrast to correlation, it is able to detect non-linear dependencies. ARACNE calculates *I*(*X*;*Y*) for every pair of gene expression profiles *X* and *Y*, and applies the data processing inequality to remove the majority of indirect dependencies. For each triplet *X*, *Y* and *Z* the weakest link is removed, e.g. the edge between *X* and *Y* is removed if *I*(*X*;*Y*)≤ min(*I*(*X*;*Z*), *M*(*Z*;*Y*))−*ε*. The tolerance threshold *ε* is used to adjust for the variance of the mutual information estimator. To generate the ARACNE based networks we used the *minet* R package [32] with the following parameters: *e* *s* *t* *i* *m* *a* *t* *o* *r*=*m* *i*.*e* *m* *p* *i* *r* *i* *c* *a* *l*, *d* *i* *s*=*e* *q* *u* *a* *l* *w* *i* *d* *t* *h* and *ε*=0.

#### MIC: maximal information coefficient

The MIC [30] is a recently proposed measure of the strength of association between two variables, closely related to mutual information. Instead of using a single discretisation strategy to bin the compared variables, it chooses individual bins for each variable, such that value of mutual information *I*(*X*;*Y*) is maximised. Compared to standard estimation of *I*(*X*;*Y*) value used in ARACNE, the optimised estimation provided by MIC is able to detect a wider range of non-linear associations. To generate MIC based networks we used the *minepy* Python library [33] with the following parameters: *α*=0.6 and *c*=15.

#### Inference of the co-expression networks counterparts

To fairly compare the co-prediction and co-expression networks generated from the same data, we had to make sure they match in size. To do that, for every co-prediction network *C* with *m* edges and *n* nodes, we created two co-expression counterparts:

- *S* *E*(*C*): co-expression network with *m* edges
- *S* *N*(*C*): co-expression network with *n* nodes

Pearson and MIC methods directly compute the pairwise similarity between the gene expressions. Given that, we generated *S* *E*(*C*) using *m* gene pairs with the highest similarity coefficient. To build *S* *N*(*C*) we used as many top gene pairs as needed, to reach at least *n* nodes (as we included all pairs tied on the similarity value, sometimes we end up with a few nodes more).

ARACNE uses a pruning procedure and generates a weighted network, not a list of pairwise similarities. When the resulting network was smaller than *m* edges or *n* nodes, we increased the default tolerance threshold *ε* to obtain a large enough network. This was the case for the *CNS* (*ε*=0.002) and the *Dlbcl* datasets (*ε*=0.043). Then we used the edge weights to select top gene pairs, as in the case of Pearson and MIC methods.

Several examples of inferred co-prediction networks and corresponding co-expression networks are visualised in Section 7 of the Additional file 1: Supplementary Material and are accompanied, in there, by an initial analysis of selected topological properties in Section 3. All generated networks are provided in the Additional file 2.

### Enrichment analysis

To understand the biological information captured by the generated networks we conducted an enrichment analysis. This is a statistical method of checking whether a set of genes have common characteristics. In our study, the set is defined by the nodes of the generated functional network and is analysed with PANTHER [34]. Because many statistical tests are performed (one for each term) at the same time, PATHER uses Bonferroni correction for multiple testing with *α*=0.05. We searched for two categories of biological knowledge: Gene Ontology (GO) terms and PANTHER pathways (176 primarily signalling pathways). From the set of GO term, we selected only the manually curated annotations that were supported by experimental evidence.

### Disease association analysis

To evaluate the predictive power of the generated networks, and to assess their relevance within a cancer-related context, we analysed the relationships between known disease-associated genes. We used two sources for the disease associations: Malacards (a meta-database of human maladies consolidated from 64 independent sources) [35] and the union of several manually curated databases (OMIM [36], Orphanet [37], Uniprot [38] and CTD [39]). A complete list of disease-associated genes is provided in the Additional file 3.

We looked at two properties: (1) the proximity of the disease-associated genes within a network and (2) the number of triangles in a network, containing one or more disease-associated genes. Higher proximity represents stronger functional relationship between genes involved in the disease. Triangles represent groups of attributes used together across different prediction rules, and therefore indicate strong mutual relationship between the genes (useful in the discovery of potential new disease associations). Triangles are also the smallest non-trivial motifs that can be found in a complex network and over-represented motifs usually identify functional units of biological processes in cells [40].

The proximity of disease-associated genes was measured using the average shortest path length (SPL). The proximity was defined as a ratio of two distances: average SPL between all pairs of the non-associated genes and average SPL between all pairs of disease-associated genes *A*:

$$ \frac{1}{n} \sum_{i=1}^{n} w_{i} \frac{\overline{SPL}(CC_{i} \setminus A)}{\overline{SPL}(A)}, \text{where } w_{i} = \frac{|CC_{i}|}{\sum_{j=1}^{n}{|CC_{j}|}} $$

As the generated networks often were disconnected (had more than 1 connected component), we introduced a weight *w*_*i*_ that represents the relative size of a connected component *C* *C*_*i*_. Components with less than 3 nodes or disease-associated genes were not used in the calculation.

## Results

The main results described in this section are based on the analysis of 8 real-world datasets. The only exception is the subsection below, which reports the test results on synthetic datasets.

### Identification of predefined relationships in synthetic datasets

To verify how well FuNeL is able to identify functional relationships, we tested it first on synthetic datasets generated using GAMETES. We used 80 different model configurations that varied in heritability, number of SNPs and ease of detection, and tested the success rate on 50 datasets per model. Given the small number of attributes in the synthetic datasets, we used only the *C*_2_ protocol configuration in the tests (no feature selection, single learning phase). The percentage of successfully identified relationships for each model is reported in Table 3. We counted as success the presence of an edge between the interacting pair of SNPs in the inferred network.

**Table 3:** FuNeL success rate in identification of disease-predicting SNPs

	5 SNP		10 SNP		15 SNP		20 SNP		25 SNP	
Her.	L-EDM	H-EDM	L-EDM	H-EDM	L-EDM	H-EDM	L-EDM	H-EDM	L-EDM	H-EDM
0.001	6 %	16 %	8 %	18 %	4 %	10 %	4 %	12 %	12 %	16 %
0.005	8 %	82 %	0 %	86 %	6 %	80 %	2 %	82 %	8 %	72 %
0.01	8 %	96 %	8 %	100 %	8 %	100 %	12 %	100 %	14 %	100 %
0.05	14 %	100 %	60 %	100 %	42 %	100 %	34 %	100 %	34 %	100 %
0.1	100 %	100 %	100 %	100 %	100 %	100 %	100 %	100 %	100 %	100 %
0.2	100 %	100 %	100 %	100 %	100 %	100 %	100 %	100 %	100 %	100 %
0.3	100 %	100 %	100 %	100 %	100 %	100 %	100 %	100 %	100 %	100 %
0.4	100 %	100 %	100 %	100 %	100 %	100 %	100 %	100 %	100 %	100 %

The datasets differed with respect to heritability, number of SNPs and detection difficulty (L-EDM models were the hardest, H-EDM the easiest)

As expected a higher success rate was obtained for models where relationships were easy to detect (H-EDM). The performance increased with higher values of heritability and 100 % success rate was obtained for heritability values above 0.05 regardless of model difficulty. The overall results are similar to those reported in [28], or even slightly better, as FuNeL’s success rate was unaffected by the increase in the number of SNPs.

### Complementarity of the enriched terms

To test how unique are the biological terms (GO terms and pathways) over-represented in the inferred FuNeL networks, we measured an overlap between terms found for each type of network. We defined the overlap between terms enriched for networks inferred using configurations *C*_*a*_ and *C*_*b*_ as:

$$ O(C_{a},C_{b}) = \frac{c}{u_{a} + u_{b} + c} $$

where *c* is the number common terms, *u*_*a*_ is the number of unique terms for *C*_*a*_ and *u*_*b*_ is the number of unique terms for *C*_*b*_ (see Additional file 4 for a complete list of enriched terms).

Table 4 summaries the pair-wise overlap between the 4 different FuNeL configurations. For GO terms we reported the average overlap between the: biological process, cellular component and molecular function categories. Although configurations that operate on the same dataset (*C*_1_– *C*_3_ and *C*_2_– *C*_4_) shared the most terms/pathways, the overlap is quite far from 100 %. Thus the remaining difference is a result of the second training stage. Configurations used on different datasets (i.e. different set of attributes) resulted in networks sharing less than 40 % GO terms and 20 % pathways.

**Table 4:** Average overlap of enriched GO terms and pathways between different FuNeL configurations. The overlap was averaged across all 8 datasets

	Gene Ontology				Pathways			
	*C*_1_	*C*_2_	*C*_3_	*C*_4_	*C*_1_	*C*_2_	*C*_3_	*C*_4_
*C*_1_	—	0.353	0.749	0.405	—	0.186	0.513	0.183
*C*_2_		—	0.321	0.701		—	0.095	0.591
*C*_3_			—	0.364			—	0.104
*C*_4_				—				—

Similarly, we analysed the term overlap between co-prediction and co-expression by comparing the *C*_*i*_ networks with their co-expression counterparts *SE*(*C*_*i*_) and *SN*(*C*_*i*_) generated with different approaches (see Table 5). We found the percentage of overlap to be similar across the different inference methods. The overlap in enriched terms was never higher than 62 % (still leading to a difference around 40 %) and was the largest for configuration not using feature selection (*C*_2_ and *C*_4_). In general the percentages were lower for biological pathways with a minimum of only 10 % of shared terms. Low values of terms overlap indicate that the co-prediction and the co-expression approaches can be seen as complementary. Despite starting from the same dataset, they generate networks expressing different biological information.

**Table 5:** Average overlap of enriched GO terms and pathways between the co-prediction and co-expression networks

		Co-expression (SE)				Co-expression (SN)			
Method	Cat.	*C*_1_	*C*_2_	*C*_3_	*C*_4_	*C*_1_	*C*_2_	*C*_3_	*C*_4_
Pearson	GO	0.280	0.414	0.297	0.432	0.315	0.576	0.367	0.488
	path.	0.223	0.260	0.258	0.190	0.264	0.400	0.175	0.287
ARACNE	GO	0.348	0.621	0.272	0.565	0.333	0.612	0.277	0.535
	path.	0.126	0.463	0.139	0.479	0.085	0.423	0.016	0.356
MIC	GO	0.316	0.513	0.283	0.487	0.300	0.614	0.289	0.527
	path.	0.097	0.339	0.142	0.315	0.112	0.469	0.080	0.352

Each co-expression network *C*_*i*_ was compared to the corresponding co-expression networks *S* *E*(*C*_*i*_) and *S* *N*(*C*_*i*_). The overlap was averaged across all 8 datasets

### Quantifying the amount of captured biological knowledge

The amount of biological knowledge (number of enriched terms) captured by a network is related to its size (number of nodes). To fairly compare the networks of different sizes we used the normalised Enrichment Score (ES):

$$ ES=\frac{number\ of\ enriched\ terms}{number\ of\ nodes} $$

The score assesses if a network contains biologically related nodes. The higher it is, the larger is a biological similarity between the nodes of a network. All computed score values are available in the Additional file 5.

To have a global view of the performances of each inference method in term of ES, we performed a two-step analysis for each enrichment category. First, using the ES, we ranked the networks generated by each method in order to identify the best performing one. See Section 4 of the Additional file 1: Supplementary Material for the complete analysis.

Once we identified the best network for each method, we ranked them together by ES and calculated their average rank across the datasets. The results of this analysis are reported in Table 6. MIC performed best when ES was calculated using the GO terms (it was ranked first in each of those categories). When ES was calculated using the biological pathways, *C*_4_ and ARACNE *S* *E*(*C*_1_) shared the highest rank.

**Table 6:** Average ranks based on the Enrichment Score for the best performing networks of each inference method

Category	FuNeL	Pearson	ARACNE	MIC
GO BP	C4 (3)	**SE(C3) (1.5)**	SN(C3) (4)	**SN(C3) (1.5)**
GO MF	C3 (3.5)	SN(C3) (3.5)	SN(C3) (2)	**SN(C3) (1)**
GO CC	C3 (4)	SN(C1) (3)	SN(C3) (2)	**SN(C3) (1)**
Patwhays	**C4 (1.5)**	SN(C2) (3.5)	**SE(C1) (1.5)**	SE(C3) (3.5)
Average	3	2.88	2.38	**1.75**

For each category and for each method, we report the network used in the analysis. The ranks (in brackets) were averaged across all 8 datasets, and the highest ranks are shown with bold font. The last row reports the average ranks across all the biological categories. The following abbreviations were used for GO categories: biological process (BP), molecular function (MF) and cellular component (CC)

Table 6 shows that the best performing networks for each method were mostly *C*_3_ co-expression counterparts, in particular *S* *N*(*C*_3_). This is consistent with the result of the topological analysis in Section 3 of the Additional file 1: Supplementary Material were these networks were found to have the lowest number of nodes, and suggests that smallest networks tend to be more enriched. The difference in performance between the FuNeL configurations is mainly a result of the application of the second machine learning phase (the best networks were *C*_3_ and *C*_4_).

In Additional file 1: Table S9 we reported the results of a similar analysis where we compared the similarity-based inference methods against FuNeL (ranks in there range from 1 to 12: 4 *C*_*i*_ + 4 *SE*(*C*_*i*_) + 4 *SN*(*C*_*i*_). In this pairwise analysis, FuNeL networks performed similarly to Pearson and ARACNE. We did not observe any consistent winner across all the enrichment categories. MIC seems to have better results than FuNeL only for GO categories, as emerged from Table 6, while FuNeL networks tend to be more enriched for biological pathways.

### Evaluation of the networks in a disease context

To verify if the topology of the inferred networks is biologically meaningful, we analysed how it defines the relationships between genes that are known to be associated with a disease targeted by each dataset. We expected the disease-associated genes to be more closely connected than other genes and to be present in functional units, such as triangle motifs. We measured the proximity of the disease-associated genes (i.e. how closely connected they are compared with non-disease-associated genes) and counted the number of triangular relationships present in each network (i.e. the percentage of triangles containing one, two or three disease-associated genes). We repeated the two-step analysis as presented in Section *Quantifying the amount of captured biological knowledge* by using the gene-disease metrics for the ranking. The results are reported in Table 7. The detailed results for each inference method are available in Section 5 of the Additional file 1: Supplementary Material.

**Table 7:** Average ranks based on the disease-associations for the best performing networks of each inference method

Source	Category	FuNeL	Pearson	ARACNE	MIC
Curated	1A	**C2 (1)**	SN(C2) (4)	SN(C3) (2.5)	SN(C2) (2.5)
	2A	**C3 (1)**	SN(C3) (2)	SE(C2) (3)	SN(C2) (4)
	3A	C1 (2)	SN(C1) (3)	SE(C4) (4)	**SE(C2) (1)**
	Proximity	**C2 (1)**	SN(C3) (2.5)	SE(C4) (2.5)	SE(C2) (4)
	Average	**1.25**	2.88	3	2.88
Malacards	1A	**C2 (1)**	SN(C2) (4)	SN(C4) (3)	SE(C4) (2)
	2A	**C2 (1.5)**	SN(C4) (4)	**SE(C4) (1.5)**	SN(C2) (3)
	3A	C3 (2)	SN(C4) (3)	SE(C2) (4)	**SN(C2) (1)**
	Proximity	**C2 (1)**	SE(C4) (4)	SE(C4) (3)	SE(C2) (2)
	Average	**1.78**	3.75	2.88	2

For each category and for each method we report the network used for the analysis. The ranks (in brackets) were averaged across all 8 datasets, and the highest ranks are shown with bold font. The *Average* row reports the average ranks across all the categories. The number of disease-associated genes participating in a triangle is denoted as 1A, 2A and 3A

The average ranks, for both sources of disease associations, suggest that co-prediction outperforms the other inference paradigms. The proximity of the disease-associated genes was in general higher in *C*_2_ network. Therefore, the co-prediction paradigm has identified the core elements of the network more accurately. This result highlights the benefits of including functional information, whenever these are available, in the network inference process (FuNeL is using the class labels assigned to the samples of the dataset), in contrast to the co-expression approach solely based on gene expression similarity (unsupervised).

There is also a clear difference in the number of disease-associated genes participating in the triangles; co-prediction networks were ranked higher than the co-expression networks. The only category in which MIC had a higher rank was 3*A*. However, considering that there were not many triangles with disease-associated genes, many ties affected the ranks in this category. Overall, these results demonstrate the higher predictive potential of the FuNeL networks in identifying new disease associations.

### Prostate cancer case study: enriched terms

To compare in detail the difference in biological knowledge captured by the co-prediction and co-expression networks, we followed our global analysis with a case study focused on a dataset targeting a single disease — prostate cancer [41]. We were especially interested in specific knowledge captured by one paradigm but not the other.

**Fig. 4:**
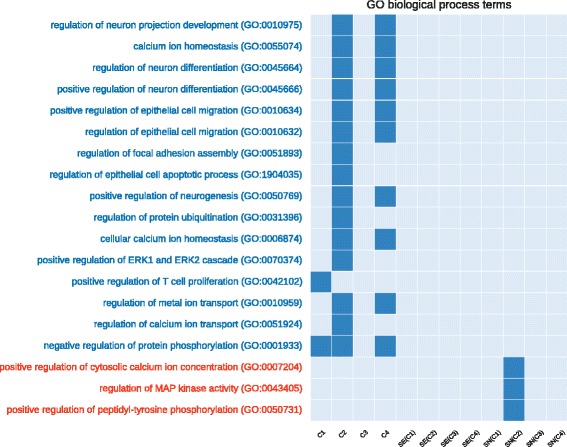
Number of unique enriched GO terms (biological process) for each network configuration (generated from the prostate cancer dataset). On the x-axis we show the 12 investigated networks. On the y-axis we show the names of enriched terms unique to co-prediction or Pearson co-expression networks. *Red* terms are associated with co-expression networks, *blue* with co-prediction. *Empty columns* indicate networks with no unique terms

**Fig. 5:**
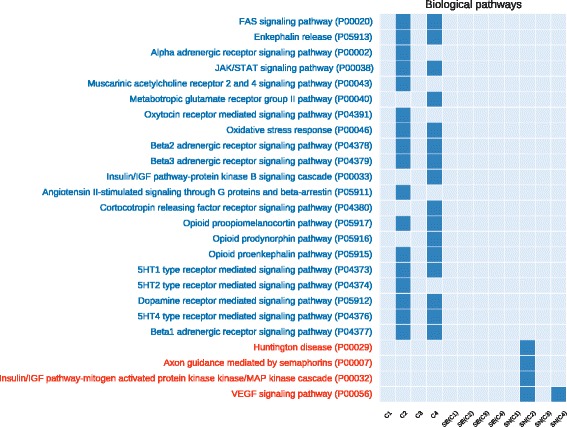
Number of unique enriched biological pathways for each network configuration (generated from the prostate cancer dataset). On the x-axis we show the 12 investigated networks. On the y-axis we show the names of enriched terms unique to co-prediction or Pearson co-expression networks. *Red* terms are associated with co-expression networks, *blue* with co-prediction. *Empty columns* indicate networks with no unique terms

In Figs. 4 and 5 we compared the co-prediction and Pearson co-expression networks inferred from the prostate cancer dataset. We focused on unique GO terms and pathways, enriched only in one type of networks. For the sake of readability we filtered out the generic GO terms (with depth <9 in the GO hierarchical structure). *C*_2_ was the network with the largest number of unique terms, followed by *C*_4_ and *S* *N*(*C*_2_). We found 16 GO terms and 21 pathways unique to co-prediction networks and only 3 GO terms and 4 pathways unique to co-expression networks. A similar disproportion in favour of the co-prediction networks was found in comparison with MIC and ARACNE networks (see Additional file 1: Figures S2 and S3).

We found several of the unique GO terms enriched in the co-prediction networks to be related to prostate cancer. The role of the *Protein ubiquination* in prostate cancer was recently analysed and showed an impact for its treatments [42]. *ERK* pathway is involved in the motility of prostate cancer cells [43]. Prostate cancer cells seems to alter the nature of their *calcium* influx to promote growth and acquire *apoptotic* resistance [44]. Furthermore, the role of *calcium homeostasis* in the majority of the cell-signaling pathways involved in carcinogenesis has been well established, prostate cancer included [45].

A number of enriched pathways specific to co-prediction networks are also highly relevant to the prostate cancer. Several studies demonstrated the involvement of the *JAK/STAT pathway* in the prostate cancer development [46, 47]. There is multiple evidence suggesting that one of the major aging-associated influences on prostate carcinogenesis is *oxidative stress* and its cumulative impact on DNA damage [48, 49]. Finally, *FAS* (also called Apo1 or CD95) plays a central role in the physiological regulation of programmed cell death and has been implicated in the pathogenesis of various malignancies and diseases of the immune system including prostate cancer [50].

We also performed an additional analysis of the biological terms related to the hubs (highly connected nodes) of the inferred networks. A node *v* was considered to be a hub if its degree was at least one standard deviation above the mean network degree. To compare the networks, we used the 10 most frequent Gene Ontology terms (biological processes with at least depth 10) shared among each network’s hubs. When considering Pearson inference approach for co-expression we found 16 unique terms for co-prediction networks, 19 unique terms for co-expression networks and 11 common terms. The results further highlight biological terms exclusively associated either with co-prediction and co-expression networks. The complete analysis (method by method) is available in the Additional file 1: Supplementary Material (Figures S4, S5 and S6).

**Fig. 6:**
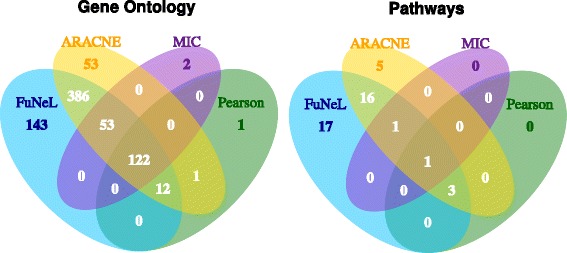
Overlap of enriched terms between the best performing networks in the disease-association analysis (curated databases). On the left the overlap of GO terms (including all 3 categories: BP, CC and MF), on the right the overlap of pathways

A further analysis of term overlap was conducted using only the best performing networks in the curated disease-association analysis (namely *C*_2_ for FuNeL, *S* *N*(*C*_3_) for Pearson, *S* *E*(*C*_4_) for ARACNE and *S* *E*(*C*_2_) for MIC, see Section 5 of the Additional file 1: Supplementary Material for details). In Fig. 6 we show the overlap of GO terms (including all three GO categories) and pathways across networks from different inference algorithms. In both categories FuNeL had much larger number of unique terms than the co-expression methods and it shared the largest number of terms with ARACNE. In total 122 common GO terms were found between all the methods, while there was only 1 common pathway. Figure 6 further highlights the complementarity between the co-prediction and co-expression approaches in terms of captured biological knowledge.

### Prostate cancer case study: disease associations

We searched the literature and the public cancer databases (not used in the inference process), to verify if key nodes in the generated networks are associated with prostate cancer. As a measure of node importance we used the node degree (number of connections) and the betweenness centrality (number of shortest paths between all pair of nodes pass through a given node).

**Literature analysis**

We picked the top 3 most connected nodes (hubs) for each of the four co-prediction networks. The set contained six genes: *GSTM2*, *NELL2*, *CFD*, *PTGDS*, *PAGE4* and *LMO3*. All the genes from this set, except *LMO3*, were also found to be the most central nodes (with highest betweenness centrality).

Almost all these genes are related with prostate cancer:

- *NELL2* contributes to alterations in epithelial-stromal homeostasis in benign prostatic hyperplasia and codes for a novel prostatic growth factor [51], and is also an indicator of expression changes in cancer samples [52],
- *CFD* (adipsin gene) is over expressed in PP periprostatic adipose tissue of prostate cancer patients [53],
- *PTGDS* (and other 2 genes) are expressed at consistently lower levels in clinical prostate cancer tissues and form a signature that predicts biochemical relapse [54],
- *PAGE4* modulates androgen receptor signaling, promoting the progression to advanced lethal prostate cancer [55], and has a significantly lower expression level in patients with prostate recurrent disease [56],
- *LMO3* interacts with *p53*, a well known gene tumour suppressor in prostate cancer [57].

The only gene without literature support was *GSTM2*. It might represent a good target for further experimental verification.

**Validation on independent data**

To further validate the biological significance of the inferred networks, we used an independent prostate cancer dataset [58] from the cBioPortal for Cancer Genomics [59]. We analysed the top 10 hubs (nodes with highest degree) and the top 10 central nodes (with highest betweenness centrality) in the co-prediction network that better performed in the gene-disease association analysis using the curated databases: *C*_2_ (see Additional file 1: Table S14). The genes with highest degree were: *PTGDS*, *PAGE4*, *NELL2*, *GSTM2*, *PARM1*, *MAF*, *LMO3*, *COL4A6*, *RBP1* and *ABL1*. For the betweenness centrality, the set was almost identical, only *RBP1* was replaced by *MYH11*. On average the expression in samples was altered in 31.8 % cases for hubs and in 35.6 % cases for central nodes. The most altered genes were found to be downregulated at the mRNA level: *COL4A6* (65 %), *MYH11* (58 %), *PARM1* (53 %) and *GSTM2* (52 %). In addition, genomic alterations in several key genes have been found to be strongly co-occurent (e.g. *PTGDS* – *GSTM2*, *PAGE4* – *COL4A6*, *PAGE4* – *RBP1*, etc.).

**Fig. 7:**
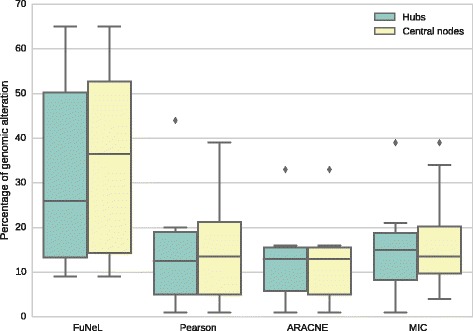
Distribution of the percentage genomic alteration in the samples of an independent dataset for top 10 hubs and central nodes. The topologically important genes were selected from the best performing networks in the disease-association analysis on curated datasets

When we repeated this analysis for the co-expression networks that were best ranked in the gene-disease analysis using the curated databases (*S* *N*(*C*_3_) for Pearson, *S* *E*(*C*_4_) for ARACNE and *S* *E*(*C*_2_) for MIC), we found that on average the alteration level was consistently lower, at most half of the co-prediction key genes. The percentages of alterations are represented as boxplots in Fig. 7, while the average alterations are reported in Table 8. As Fig. 7 shows, our method is able to identify many more genes with higher percentage of alteration than other methods. Therefore, the topologically important nodes in the best co-prediction network represent genes more strongly related to the prostate cancer, with over two times more frequent genomic alterations.

**Table 8:** Average percentage of genomic alteration for top hubs and central nodes in the independent dataset

Genes	FuNeL	Pearson	ARACNE	MIC
Hubs	31.8 %	14.2 %	12.3 %	15.2 %
Central nodes	35.6 %	14.7 %	12.2 %	17.1 %

The detailed list of genomic alterations for top 10 hubs and top 10 central nodes for each analysed network is shown in Section 6 of the Additional file 1: Supplementary Material (Figures S7–S14).

## Discussion

We proposed FuNeL, a protocol to infer functional networks based on the *co-prediction* paradigm where the structure of a rule-based machine learning model (in this paper the rules of a classification algorithm called BioHEL) is used to identify relationships between genes. We tested FuNeL on synthetic datasets and obtained a high success rate in identifying pairwise relationships between attributes. Encouraged by this result, we hypothesised that a rule-based machine learning model, with its complex knowledge representation, might be used to identify biologically meaningful relationships that escape the standard inference methods.

To test this hypothesis, we evaluated 4 different configurations of the inference protocol using 8 cancer-related transcriptomics datasets. We compared FuNeL with other 3 co-expression inference methods by using networks of matching size generated from the same data. We looked at the differences, between co-prediction and co-expression, from three points of view: basic topological properties, enriched biological terms and relationships between known disease-associated genes.

The comparison of networks topology (see Section 3 of the Additional file 1: Supplementary Material) revealed the influence of the protocol options. Not surprisingly, both the feature selection and the second training phase reduced the size of the networks, but at the same time, increased the clustering coefficient and the number of connections. The clustering coefficient was found to be lower in almost all the ARACNE networks, probably due to the pruning procedure, it was also lower in many MIC networks. Moreover, when feature selection was applied, the resulting networks had higher clustering coefficient than Pearson co-expression networks with the same number of edges. Interestingly, all co-expression networks were less compact, with up to 3 times higher diameter for Pearson and ARACNE and up to 7 times higher for MIC.

The differences in networks topology translated to differences in contained biological information. The overlap between enriched GO terms and pathways across protocol configurations was generally low, indicating that different configurations infer networks that capture different biological knowledge. The same terms overlap between the co-prediction networks and their equivalent co-expression counterparts was even lower, never exceeding 62 %. We interpret that as evidence, that the biological knowledge captured by the two paradigms is not completely redundant, but in a large part complementary.

The most apparent differences between the networks were observed during the analysis of the connections between genes known to be related to a specific disease. The disease-associated genes were more closely connected (higher proximity) in the co-prediction networks, which means that the disease-related nodes of the network were closer to its core. We also found that the number of functional units (triangle motifs), that can identify new gene-disease associations, was higher in the co-prediction networks. Therefore, we conclude that the co-prediction networks better capture the abstract concept of functional relationship.

The prostate cancer case study further confirmed this conclusion. We found enriched GO terms and biological pathways, unique to the co-prediction networks, to be reported in the literature as related to prostate cancer. Furthermore, FuNeL generated networks enriched with knowledge totally missed by all the co-expression networks when using the prostate cancer dataset. We also found that genes corresponding to the topologically important nodes in the co-prediction networks: (1) were altered in a high percentage of tumour samples in an independent cancer transcriptomic study, and (2) were already associated with prostate cancer according to the specialised literature. Therefore, the co-prediction networks not only capture biological knowledge complementary to the co-expression networks, but also highlights better the important genes involved in the disease process.

The superior performance of FuNeL networks in identifying the disease-associated genes is likely a result of effective use of the class labels of the samples, which the similarity-based methods ignore. Although it would be tempting to attribute this performance difference entirely to the use of supervised learning in FuNeL, it would be an overstatement, as the knowledge of explicit links between genes and diseases is not available to it in training. Our hypothesis is that this is rather a result of differences in expression values of the disease-associated genes, which taken together are able to discriminate between sample phenotypes.

Given that our co-prediction networks were found to be not only biologically meaningful, but also complementary to similarity-based functional networks, we believe that network inference based on machine learning models deserves to be studied in more detail in the future. In here we only touched the subject of feature selection and network post-processing, and although we now know they indeed influence the network topology and its biological interpretation, there are many strategies to choose from in that respect.

At the same time, the machine learning step in the FuNeL protocol does not have to be limited to the rule-based machine learning methods. We can imagine unsupervised methods, such as the Apriori algorithm for association rule learning, or other supervised methods, such as decision tree algorithms (e.g. C4.5 or random forest), replacing BioHEL in the FuNeL protocol. Some adjustment would be necessary to extract the knowledge from a different model representation, but the rest of the protocol could remain unchanged. For example in the case of the decision trees, relationships could be inferred between attributes that share the same path from the root to the leaves of a tree. This potential flexibility in the choice of a learning algorithm, together with the ability to apply the protocol to different types of data, becomes important in the context of results correctness. As has been discussed to a great length in [60], when methods or data used in the network inference process are tightly controlled, some results will replicate more easily than others not because they are correct, but due to a replicable bias. Therefore a diversity in methods and data is a necessary condition to be able to converge on the scientific truth.

Finally, in terms of testing new functional networks, there is a limit of how thorough and complete a manual literature analysis can be, which leads to a great need of synthetic or experimentally validated benchmarks, similar to those proposed for protein-protein interaction networks or gene regulatory networks. Although we understand that this would be a difficult and challenging task, we see this as a necessary step on the way to refining the functional inference methods.

## Conclusions

We presented FuNeL: a protocol for the inference of functional networks from rule-based machine learning models. FuNeL is based on the co-prediction paradigm, which hypothesises that genes used together with a rule-based machine learning model, are more likely to be functionally related. We verified that FuNeL correctly identifies relationships in synthetic datasets and we thoroughly compared FuNeL to three co-expression inference methods: Pearson correlation coefficient, ARACNE and MIC, on 8 real-world datasets. We contrasted the different approaches by looking at the inferred networks topology, enriched biological terms and the relationships between genes associated with cancer. We found that FuNeL networks capture relevant biological knowledge that is complementary to what is captured by the co-expression approaches, and demonstrated that FuNeL networks are better at identifying relationships between genes with known disease associations.

In future works we will explore the extension of the protocol with methods that combine topology and biological enrichment [61, 62]. We would also like to test FuNeL using different machine learning algorithms. As previously discussed, FuNeL is a flexible protocol in which the machine learning algorithm can be easily interchanged. Therefore, we will try to assess the impact of different learning algorithms in the inference of functional networks.

## Additional files

Additional file 1 Supplementary Material. Supporting information and detailed results referenced in the main text. (PDF 5550 kb)

Additional file 2 Networks. All networks used in the analysis given as a list of edges. (XZ 11980 kb)

Additional file 3 Disease-associated genes. Complete list of the disease-associated genes for each dataset. (XZ 46 kb)

Additional file 4 Enriched terms. Complete list of enriched terms for each dataset and network. (XZ 3041 kb)

Additional file 5 Enrichment Score. Complete list of Enrichment Score values for each dataset and network. (XZ 4 kb)
