# Supplementary material for: Functional networks inference from rule-based machine learning models
Source: BioData Min. 2016 Sep 5;9:28. doi: 10.1186/s13040-016-0106-4 (PMC5011349; doi:10.1186/s13040-016-0106-4)
Supplement: Supplementary file 1 — Supplementary Material. Supporting information and detailed results referenced in the main text. (PDF 5550 kb) [file 13040_2016_106_MOESM1_ESM.pdf]

Supplementary Material

Functional networks inference from machine learning models

Nicola Lazzarini, Paweł Widera, Stuart Williamson, Rakesh Heer, Natalio Krasnogor and Jaume Bacardit

1 Classification accuracy under feature selection

To choose the default percentage of attributes retained in the feature selection procedure, we performed a preliminary analysis using all 8 transcriptomic datasets from the main article. We evaluated how the BioHEL classification accuracy changes with the number of selected features (using linear SVM-RFE). The accuracy was measured using a standard 10 cross-fold validation. The full experiment (not reported here) used $100\%, 90\%, 80\%, ..., 10\%$ of the original dataset attributes.

We found that even when only 10% of the attributes are retained, the classification accuracy remains almost unchanged. Specifically, with 10% of the original attributes the accuracy increased for 2 datasets, slightly decreased for 3 datasets and remained unaltered for the other datasets. The exact results are reported in Table S1 below:

Table S1: BioHEL classification accuracy for each dataset, in 10-fold cross-validation experiments on the original and reduced set of attributes (before and after the feature selection). Linear SVM-RFE was used to select best 10% of the attributes.

**dataset**	**all attributes**	**10% attributes**
Dlbcl	0.871	0.886
CNS	0.473	0.451
Leukemia	0.945	0.945
Lung-Michigan	0.980	0.980
Lung-Harvard	0.978	0.964
Prostate	0.892	0.892
AML	0.637	0.592
Colon-Breast	0.903	0.940

Given that we were able to maintain good classification accuracy despite large reduction in number of used attributes, we decided to use 10% attributes as a default setting for the FuNeL feature selection procedure. However, this FuNeL parameter is under the user control and the default setting can be changed.

2 Time complexity

The FuNeL protocol has four stages (see Figure 2 in the main article): (1) feature selection (optional), (2) rule-based network generation, (3) permutation test and (4) second rule-based network generation (optional).

The running time for the whole pipeline depends on the rule set generation time (execution time of BioHEL), as the optional feature selection stage can be seen as running in constant time. Two main factors that influence the rule set generation time are: (1) the **number of attributes** and (2) the **number of samples**.

We performed an execution time analysis of BioHEL using the largest (in terms of number of attributes) Colon-Breast dataset (Chowdary *et al.*, 2006). In the feature selection stage we retained: 20, 200, 2000, 10 000 and 20 000 attributes. From each of these 5 datasets we generated 100 random subsets of 50, 40, 30, 20 and 10 samples. Finally, we ran BioHEL 1000 times to obtain 1000 rule sets for each dataset.

Figure S1 shows the running times averaged across 100 000 runs (1000 runs for each of the 100 datasets).

The total execution time of FuNeL configurations C_1 and C_2 is calculated as:

$$T_1 = (rule\_sets \times t(atts_1, samples)) + (permutation\_runs \times t(atts_1, samples)) \quad (1)$$

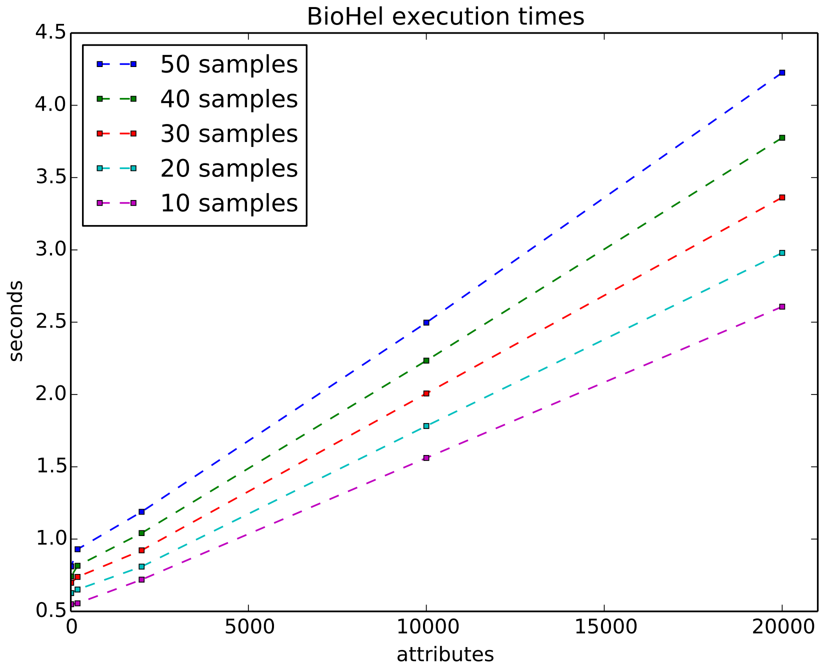

Figure S1: Average execution times of a single BioHEL run for a given number of samples and attributes.

where $rule\_sets$ is the number of inferred rule sets, $permutation\_runs$ is the number of randomised datasets used in the permutation test and $t(atts_1, samples)$ represents execution time of a single BioHEL run, that linearly depends on the size of a dataset measured in number of attributes and samples.

Configurations C_3 and C_4 require an additional run of BioHEL (step 4), and their total execution time is:

$$T_2 = T_1 + (rule\_sets \times t(atts_2, samples)) \tag{2}$$

where $atts_2$ is the number of attributes after the permutation test ($atts_1 \leq atts_2$).

It is important to notice that each run of BioHEL is independent, thus the generation of the rule sets can be trivially parallelised without any extra overhead. Given n computational cores, the total execution times could be reduced to:

$$T_{real_1} = \frac{T_1}{n} \qquad T_{real_2} = \frac{T_2}{n} \tag{3}$$

3 Comparison of networks topological properties

The network topology refers to the spatial arrangements of its elements. The analysis of topological properties tells us how different nodes are connected to each other and how their communication paths look like. There are many aspects and characteristics that can be evaluated in a network. For simplicity we report just four metrics: number of nodes, number of edges, clustering coefficient and diameter.

The *clustering coefficient* is a measure of degree to which nodes in a network tend to cluster together. It expresses the likelihood that any two nodes with a common neighbour are themselves connected. The *diameter* indicates the maximum distance between two nodes in the network.

We compared the topology of the networks built with two different approaches: co-prediction and co-expression. For each generated network we calculated the topological properties described above. We report the comparison of FuNeL networks with co-expression networks inferred with different methods. The results considering Pearson are reported in Table S2, ARACNE is reported in Table S3 while MIC analysis is shown in Table S4.

Table S2: Topological properties for co-prediction and Pearson co-expression networks generated for all 8 datasets.

		**Co-prediction**				**Co-expression (SE)**				**Co-expression (SN)**			
**Dataset**	**Cat.**	C_1	C_2	C_3	C_4	$SE(C_1)$	$SE(C_2)$	$SE(C_3)$	$SE(C_4)$	$SN(C_1)$	$SN(C_2)$	$SN(C_3)$	$SN(C_4)$
**Leukemia**	**Nodes**	421	1480	294	988	683	873	843	941	422	1482	293	979
	**Edges**	1529	2294	2154	2646	1529	2294	2154	2646	680	7145	409	2870
	**Clust.Coef.**	0.712	0.155	0.589	0.33	0.333	0.348	0.354	0.341	0.323	0.388	0.303	0.344
	**Diameter**	5	6	4	6	24	18	19	22	16	18	9	20
**LungH**	**Nodes**	429	1419	382	1030	578	930	955	1214	432	1413	384	1027
	**Edges**	1068	2317	2398	3410	1068	2317	2398	3410	617	4302	476	2650
	**Clust.Coef.**	0.344	0.298	0.43	0.404	0.356	0.373	0.376	0.372	0.341	0.386	0.296	0.376
	**Diameter**	5	8	5	7	10	23	23	21	6	22	6	23
**LungM**	**Nodes**	91	919	48	247	76	280	59	119	90	915	50	248
	**Edges**	134	1858	78	410	134	1858	78	410	224	13574	64	1510
	**Clust.Coef.**	0.379	0.262	0.418	0.457	0.465	0.525	0.446	0.514	0.539	0.523	0.493	0.511
	**Diameter**	3	5	3	3	6	11	6	6	5	14	6	12
**CNS**	**Nodes**	501	4257	494	3538	945	2152	1616	2607	501	4261	488	3532
	**Edges**	4302	25069	12769	40840	4302	25069	12769	40840	1553	171052	1502	90395
	**Clust.Coef.**	0.743	0.255	0.521	0.302	0.354	0.389	0.367	0.400	0.346	0.427	0.35	0.421
	**Diameter**	4	7	4	6	21	15	23	13	12	13	14	12
**Dlbcl**	**Nodes**	201	1699	201	1617	207	1411	1238	1790	200	1699	200	1614
	**Edges**	848	10471	7351	33170	848	10471	7351	33170	832	24280	832	17865
	**Clust.Coef.**	0.872	0.574	0.642	0.453	0.508	0.438	0.411	0.51	0.501	0.504	0.501	0.481
	**Diameter**	3	5	3	5	2	16	17	14	2	14	2	15
**GSE2191**	**Nodes**	890	4802	846	3561	837	1848	1239	1750	897	4799	839	3553
	**Edges**	3290	13424	6469	12074	3290	13424	6469	12074	3711	90410	3292	47806
	**Clust.Coef.**	0.488	0.082	0.317	0.291	0.377	0.409	0.394	0.4	0.382	0.415	0.377	0.417
	**Diameter**	5	9	5	9	25	23	19	21	21	13	25	15
**GS3726**	**Nodes**	668	2077	524	1170	879	1300	992	1367	759	2300	1739	3440
	**Edges**	1761	3255	2051	3502	1761	3255	2051	3502	1471	9808	5479	26761
	**Clust.Coef.**	0.134	0.0077	0.307	0.109	0.226	0.23	0.223	0.233	0.213	0.287	0.254	0.346
	**Diameter**	8	10	7	8	20	26	20	25	26	15	19	15
**Prostate**	**Nodes**	938	4290	704	2277	356	543	322	448	920	4298	702	2287
	**Edges**	3796	10175	3090	6546	3796	10175	3090	6546	33250	914829	16934	24427
	**Clust.Coef.**	0.328	0.245	0.29	0.25	0.565	0.607	0.541	0.584	0.655	0.703	0.641	0.711
	**Diameter**	7	10	6	8	6	9	5	8	8	11	7	12

Table S3: Topological properties for co-prediction and ARACNE co-expression networks generated for all 8 datasets.

		Co-prediction				Co-expression (SE)				Co-expression (SN)			
**Dataset**	**Cat.**	C_1	C_2	C_3	C_4	$SE(C_1)$	$SE(C_2)$	$SE(C_3)$	$SE(C_4)$	$SN(C_1)$	$SN(C_2)$	$SN(C_3)$	$SN(C_4)$
**Leukemia**	**Nodes**	421	1480	294	988	1024	1426	1356	1577	422	1480	294	989
	**Edges**	1529	2294	2154	2646	1529	2294	2154	2646	512	2416	327	1479
	**Clust.Coef.**	0.712	0.155	0.589	0.330	0.002	0.002	0.002	0.002	0.000	0.002	0.000	0.002
	**Diameter**	5	6	4	6	17	19	22	19	9	17	11	19
**LungH**	**Nodes**	429	1419	382	1030	907	1614	1653	2066	429	1419	382	1030
	**Edges**	1068	2317	2398	3410	1068	2317	2398	3410	435	1924	375	1250
	**Clust.Coef.**	0.344	0.298	0.430	0.404	0.007	0.006	0.006	0.005	0.013	0.006	0.012	0.007
	**Diameter**	5	8	5	7	23	16	15	13	14	18	10	18
**LungM**	**Nodes**	91	919	48	247	143	1321	96	370	91	920	48	247
	**Edges**	134	1858	78	410	134	1858	78	410	72	1127	34	259
	**Clust.Coef.**	0.379	0.262	0.418	0.475	0.000	0.002	0.000	0.009	0.000	0.005	0.000	0.014
	**Diameter**	3	5	3	3	13	17	11	18	11	17	5	11
**CNS**	**Nodes**	501	4257	494	3538	2002	4509	3581	5342	502	4257	494	3538
	**Edges**	4302	25069	12769	40840	4302	25069	12769	41661	513	20409	505	12358
	**Clust.Coef.**	0.743	0.255	0.521	0.302	0.004	0.005	0.006	0.026	0.004	0.005	0.004	0.005
	**Diameter**	4	7	4	6	12	8	12	7	20	9	20	12
**Dlbcl**	**Nodes**	201	1699	201	1617	380	1452	1191	2236	201	1699	201	1617
	**Edges**	848	10471	7351	33170	848	10471	7351	33890	269	14149	269	12903
	**Clust.Coef.**	0.872	0.574	0.642	0.453	0.136	0.126	0.140	0.176	0.113	0.110	0.113	0.115
	**Diameter**	3	5	3	5	13	9	11	5	12	8	12	9
**GSE2191**	**Nodes**	890	4802	846	3561	2574	5226	3846	5027	890	4802	846	3561
	**Edges**	3290	13424	6469	12074	3290	13424	6469	12076	846	10671	794	5564
	**Clust.Coef.**	0.488	0.082	0.317	0.291	0.002	0.002	0.002	0.002	0.004	0.002	0.004	0.002
	**Diameter**	5	9	5	9	19	13	16	13	30	15	30	17
**GS3726**	**Nodes**	668	2077	524	1170	1362	2167	1546	2279	668	2166	524	1170
	**Edges**	1761	3255	2051	3502	1761	3255	2051	3502	787	3250	597	1455
	**Clust.Coef.**	0.134	0.077	0.307	0.109	0.024	0.053	0.029	0.050	0.014	0.053	0.016	0.021
	**Diameter**	8	10	7	8	20	20	19	18	15	20	13	19
**Prostate**	**Nodes**	938	4290	704	2277	2760	6805	2268	4575	939	4290	704	2277
	**Edges**	3796	10175	3090	6546	3796	10175	3090	6546	1300	6095	1017	3102
	**Clust.Coef.**	0.328	0.245	0.290	0.250	0.005	0.003	0.006	0.003	0.001	0.003	0.002	0.005
	**Diameter**	7	10	6	8	13	13	15	13	12	13	9	15

Table S4: Topological properties for co-prediction and MIC co-expression networks generated for all 8 datasets.

		**Co-prediction**				**Co-expression (SE)**				**Co-expression (SN)**			
**Dataset**	**Cat.**	C_1	C_2	C_3	C_4	$SE(C_1)$	$SE(C_2)$	$SE(C_3)$	$SE(C_4)$	$SN(C_1)$	$SN(C_2)$	$SN(C_3)$	$SN(C_4)$
**Leukemia**	**Nodes**	421	1480	294	988	640	807	780	896	421	1480	294	989
	**Edges**	1529	2294	2154	2646	1529	2294	2155	2647	749	6173	432	3096
	**Clust.Coef.**	0.712	0.155	0.589	0.330	0.162	0.182	0.180	0.179	0.138	0.180	0.127	0.175
	**Diameter**	5	6	4	6	18	29	27	29	10	17	8	18
**LungH**	**Nodes**	429	1419	382	1030	384	685	703	944	429	1419	382	1030
	**Edges**	1068	2317	2398	3410	1068	2317	2399	3410	1264	5867	1045	3841
	**Clust.Coef.**	0.344	0.298	0.430	0.404	0.349	0.308	0.305	0.302	0.339	0.282	0.343	0.305
	**Diameter**	5	8	5	7	9	13	13	17	7	18	9	19
**LungM**	**Nodes**	91	919	48	247	118	626	79	219	91	919	48	247
	**Edges**	134	1858	78	410	134	1858	78	410	93	3109	38	484
	**Clust.Coef.**	0.379	0.262	0.418	0.475	0.212	0.272	0.213	0.306	0.208	0.235	0.153	0.302
	**Diameter**	3	5	3	3	8	18	7	8	6	14	3	7
**CNS**	**Nodes**	501	4257	494	3538	1424	3104	2357	3725	501	4257	495	3538
	**Edges**	4302	25069	12769	40840	4305	25131	12771	40850	704	62208	694	36027
	**Clust.Coef.**	0.743	0.255	0.521	0.302	0.124	0.154	0.144	0.159	0.089	0.162	0.091	0.161
	**Diameter**	4	7	4	6	17	11	12	10	11	10	11	10
**Dlbcl**	**Nodes**	201	1699	201	1617	475	1140	1047	1453	203	1699	203	1617
	**Edges**	848	10471	7351	33170	848	10471	7362	33172	196	74773	196	59307
	**Clust.Coef.**	0.872	0.574	0.642	0.453	0.111	0.240	0.219	0.319	0.082	0.381	0.082	0.366
	**Diameter**	3	5	3	5	21	13	16	15	11	11	11	11
**GSE2191**	**Nodes**	890	4802	846	3561	1700	4129	2617	3883	890	4803	846	3563
	**Edges**	3290	13424	6469	12074	3299	13433	6469	12207	1380	17797	1271	10540
	**Clust.Coef.**	0.488	0.082	0.317	0.291	0.109	0.095	0.098	0.098	0.120	0.095	0.118	0.099
	**Diameter**	5	9	5	9	22	15	18	16	19	15	21	15
**GS3726**	**Nodes**	668	2077	524	1170	1271	1921	1357	1996	672	2152	526	1172
	**Edges**	1761	3255	2051	3502	1890	3261	2056	3524	852	3921	538	1705
	**Clust.Coef.**	0.134	0.077	0.307	0.109	0.110	0.100	0.104	0.100	0.126	0.099	0.121	0.117
	**Diameter**	8	10	7	8	23	29	23	28	14	24	14	24
**Prostate**	**Nodes**	938	4290	704	2277	687	839	667	773	964	4290	712	2277
	**Edges**	3796	10175	3090	6546	3981	10186	3777	8257	15928	1763794	5254	308709
	**Clust.Coef.**	0.328	0.245	0.290	0.250	0.167	0.278	0.169	0.265	0.313	0.758	0.218	0.661
	**Diameter**	7	10	6	8	7	8	7	8	7	8	7	9

When analysing FuNeL networks we observed, as expected, that configurations having feature selection (C_1 and C_3) lead to networks with a smaller number of nodes than when the original set of attributes is used (C_2 and C_4). Furthermore, the second phase of machine learning modeling (C_3 and C_4) tends to reduce the number of nodes as it uses a reduced set of attributes as input (only significant nodes and their neighbours from the first training phase), while increasing both clustering coefficient and number of edges.

When comparing FuNeL and co-expression networks we notice that the ARACNE *SE* counterparts have in general more nodes. The same patter can be found in $SE(C_2$ and $SE(C_4)$ counterparts generated with Pearson and MIC, while it's not true for the *SE*-networks based on configurations that use feature selection (C_1 and C_2). Conversely, *SN*-networks differ according to the inference method used. In fact ARACNE generated *SN* counterparts with less edges, while this is true only for $SN(C_1)$ and $SN(C_3)$ inferred with MIC and Pearson. The clustering coefficient is constantly lower in ARACNE networks than in FuNeL, this is probably due to the pruning phase operated by the method. A similar trend can be noticed for MIC networks with some exceptions (e.g Prostate $SN(C_2)$ and $SN(C_4)$). A more balanced situation occurs when FuNeL is contrasted with Pearson, in fact networks generated with feature selection (C_1 and C_3) have a lower coefficient than their co-expression counterparts. Finally a clear pattern emerge when analysing the diameter of the networks. Co-prediction networks are always more compact than co-expression counterparts having up to 3 time lower diameter for MIC and Pearson and up to 7 time lower for ARACNE.

4 Enrichment score analysis

In this section we report the network average rankings, based on the Enrichment Score, across the 8 datasets for each inferring method. The networks are ranked between 1 and N (where $N = 4$ for FuNeL and $N = 8$ for Pearson, ARACNE and MIC: 4 $SE(C_i)$ + 4 $SN(C_i)$). We considered Gene Ontology terms (biological process (BP), molecular function (MF) and cellular component (CC)) and biological pathways. The last row of each table represents the average rank across different biological categories.

Table S5: Average ranks based on the Enrichment Score across the 8 datasets for the networks generated with FuNeL.

**Cat.**	**C1**	**C2**	**C3**	**C4**
**GO BP**	3	4	2	**1**
**GO MF**	4	2.5	**1**	2.5
**GO CC**	2	4	**1**	3
**Pathways**	4	2	3	**1**
**Average**	3.25	3.125	**1.75**	1.88

Table S6: Average ranks based on the Enrichment Score across the 8 datasets for the networks generated with Pearson.

	**Pearson (SE)**				**Pearson (SN)**			
**Cat.**	C_1	C_2	C_3	C_4	C_1	C_2	C_3	C_4
**GO BP**	2	4	**1**	3	6	7	5	8
**GO MF**	8	3.5	5	6	7	3.5	**1**	2
**GO CC**	2	5	4	6	**1**	8	3	7
**Pathways**	6	5	4	3	7	**1**	8	2
**Average**	4.5	4.38	**3.5**	4.5	5.25	4.88	4.25	4.75

Table S7: Average ranks based on the Enrichment Score across the 8 datasets for the networks generated with ARACNE.

	**ARACNE (SE)**				**ARACNE (SN)**			
**Cat.**	C_1	C_2	C_3	C_4	C_1	C_2	C_3	C_4
**GO BP**	4	7	5.5	8	2	5.5	**1**	3
**GO MF**	4	8	4	7	2	6	**1**	4
**GO CC**	3	7	5	8	2	6	**1**	4
**Pathways**	**1**	4	2	6	7	5	8	3
**Average**	3	6.5	4.13	7.25	3.25	5.63	**2.75**	3.5

Table S8: Average ranks based on the Enrichment Score across the 8 datasets for the networks generated with MIC.

	MIC (SE)				MIC (SN)			
**Cat.**	C_1	C_2	C_3	C_4	C_1	C_2	C_3	C_4
**GO BP**	2	6	4	5	3	8	**1**	7
**GO MF**	**1**	6	4	7	3	8	2	5
**GO CC**	3	5.5	4	5.5	2	8	**1**	7
**Pathways**	2.5	4	**1**	5.5	8	5.5	7	2.5
**Average**	**2.13**	5.38	3.25	5.75	4	7.38	2.75	5.38

In here we also report the results of the analysis where we compared each inferring method against FuNeL using the Enrichment Score. The networks are ranked from 1 to 12: 4 C_i + 4 $SE(C_i)$ + 4 $SN(C_i)$. In Table S9 we report, for each biological category and for each network, the ranks averaged across the 8 datasets. The row-wise rank is given in brackets and the highest ranks are shown with bold font. The following abbreviations were used for GO categories: biological process (BP), molecular function (MF) and cellular component (CC).

Table S9: Average network ranks based on the Enrichment Score.

		Co-prediction				Co-expression (SE)				Co-expression (SN)			
**Method**	**Cat.**	C_1	C_2	C_3	C_4	$SE(C_1)$	$SE(C_2)$	$SE(C_3)$	$SE(C_4)$	$SN(C_1)$	$SN(C_2)$	$SN(C_3)$	$SN(C_4)$
**Pearson**	**GO BP**	6.06 (6)	7.00 (7.5)	7.00 (7.5)	5.88 (3.5)	5.88 (3.5)	5.88 (3.5)	**5.12 (1)**	5.88 (3.5)	7.06 (9)	7.75 (12)	7.12 (10)	7.38 (11)
	**GO MF**	7.81 (11)	5.38 (2)	6.19 (5)	5.62 (3)	9.12 (12)	6.50 (7.5)	6.50 (7.5)	7.38 (10)	7.19 (9)	6.25 (6)	**4.31 (1)**	5.75 (4)
	**GO CC**	4.31 (5)	11.00 (12)	4.19 (4)	9.00 (10)	3.88 (2)	6.25 (6.5)	6.25 (6.5)	8.12 (8)	**3.19 (1)**	9.38 (11)	4.06 (3)	8.38 (9)
	**Pathways**	8.12 (10.5)	4.75 (2)	8.12 (10.5)	**4.38 (1)**	6.94 (8)	6.50 (6)	6.69 (7)	5.88 (5)	7.62 (9)	5.00 (3)	8.50 (12)	5.50 (4)
**ARACNE**	**GO BP**	6.69 (7)	6.25 (5.5)	6.94 (10)	**4.75 (1)**	6.25 (5.5)	8.12 (11)	6.88 (8.5)	9.25 (12)	5.19 (3)	6.88 (8.5)	5.06 (2)	5.75 (4)
	**GO MF**	7.44 (10)	6.50 (8)	6.19 (6.5)	5.69 (3)	6.00 (5)	8.75 (12)	5.62 (2)	8.62 (11)	5.81 (4)	7.00 (9)	**4.19 (1)**	6.19 (6.5)
	**GO CC**	4.31 (4)	10.75 (12)	3.44 (3)	8.25 (8)	5.50 (5)	9.38 (10)	6.75 (7)	10.12 (11)	2.44 (2)	8.88 (9)	**2.31 (1)**	5.88 (6)
	**Pathways**	7.88 (10.5)	5.38 (3)	7.88 (10.5)	5.25 (2)	**4.88 (1)**	6.25 (6)	5.50 (4)	7.00 (8)	7.56 (9)	6.50 (7)	8.38 (12)	5.56 (5)
**MIC**	**GO BP**	7.44 (8.5)	7.88 (11)	7.44 (8.5)	6.38 (5.5)	4.12 (2)	7.00 (7)	6.00 (4)	6.38 (5.5)	4.81 (3)	9.12 (12)	**3.94 (1)**	7.50 (10)
	**GO MF**	8.06 (10)	8.50 (12)	7.19 (8)	7.75 (9)	**3.62 (1)**	6.50 (5.5)	5.12 (3)	6.75 (7)	5.31 (4)	8.12 (11)	4.56 (2)	6.50 (5.5)
	**GO CC**	5.19 (6)	11.62 (12)	4.44 (4)	10.12 (10)	4.25 (3)	7.12 (7.5)	5.12 (5)	7.12 (7.5)	3.19 (2)	10.38 (11)	**1.69 (1)**	7.75 (9)
	**Pathways**	8.75 (12)	**4.75 (1)**	7.50 (10)	5.50 (3)	6.00 (5)	6.50 (6)	5.00 (2)	6.62 (7)	7.56 (11)	7.00 (9)	6.94 (8)	5.88 (4)

5 Disease association analysis

In this section we report the network average rankings across the 8 datasets for every inferring method based on the gene-disease association properties: participation in triangular relationship and proximity. We used two sources for the disease associations: Malacards (Rappaport *et al.*, 2013) (a meta-database of human maladies consolidated from 64 independent sources) and manually curated databases (OMIM (Hamosh *et al.*, 2002), Orphanet (Orphanet, 1997), Uniprot (Magrane and Consortium, 2011) and CTD (Davis *et al.*, 2014)). The networks are ranked between 1 and N (where $N = 4$ for FuNeL and $N = 8$ for Pearson, ARACNE and MIC: 4 $SE(C_i)$ + 4 $SN(C_i)$). The number of disease-associated genes participating in a triangle is denoted as 1A, 2A and 3A. The last row of each table represents the average rank across different metrics.

5.1 Malacards

Table S10: Average ranks based on the disease-association analysis (Malacards) across the 8 datasets for the networks generated with FuNeL.

**Cat.**	**C1**	**C2**	**C3**	**C4**
**1A**	3	**1**	4	2
**2A**	4	**1**	2	3
**3A**	3	3	**1**	3
**Proximity**	3	**1**	4	2
**Average**	3.25	**1.5**	2.75	2.5

Table S11: Average ranks based on the disease-association analysis (Malacards) across the 8 datasets for the networks generated with Pearson.

	**Pearson (SE)**				**Pearson (SN)**			
**Cat.**	C_1	C_2	C_3	C_4	C_1	C_2	C_3	C_4
**1A**	8	5	6	3	4	**1**	2	7
**2A**	7	4	5	3	8	2	6	**1**
**3A**	6.5	6.5	6.5	6.5	3	2	4	**1**
**Proximity**	**1.5**	5	3	**1.5**	7	6	8	4
**Average**	5.75	5.13	5.13	3.5	5.5	**2.75**	5	3.25

Table S12: Average ranks based on the disease-association analysis (Malacards) across the 8 datasets for the networks generated with ARACNE.

	**ARACNE (SE)**				**ARACNE (SN)**			
**Cat.**	C_1	C_2	C_3	C_4	C_1	C_2	C_3	C_4
**1A**	4	8	7	5.5	2.5	2.5	5.5	**1**
**2A**	7	6	8	**1**	4	3	2	5
**3A**	5.5	**1**	5.5	2	5.5	5.5	5.5	5.5
**Proximity**	7	3	5	**1**	6	2	8	4
**Average**	5.88	4.5	6.38	**2.38**	4.5	3.25	5.25	3.88

Table S13: Average ranks based on the disease-association analysis (Malacards) across the 8 datasets for the networks generated with MIC.

	**MIC (SE)**				**MIC (SN)**			
**Cat.**	C_1	C_2	C_3	C_4	C_1	C_2	C_3	C_4
**1A**	6	3	7.5	**1**	5	2	7.5	4
**2A**	8	3	5	2	7	**1**	6	4
**3A**	7	2	8	6	5	**1**	3	4
**Proximity**	6	**1**	2	5	7	4	8	3
**Average**	6.75	2.25	5.63	3.5	6	2	6.13	3.75

5.2 Curated databases

Table S14: Average ranks based on the disease-association analysis (curated databases) across the 8 datasets for the networks generated with FuNeL.

**Cat.**	**C1**	**C2**	**C3**	**C4**
**1A**	3	**1**	4	2
**2A**	3	4	**1**	2
**3A**	**1**	2.5	2.5	4
**Proximity**	4	**1**	3	2
**Average**	2.75	**2.13**	2.67	2.5

Table S15: Average ranks based on the disease-association analysis (curated databases) across the 8 datasets for the networks generated with Pearson.

	**Pearson (SE)**				**Pearson (SN)**			
**Cat.**	C_1	C_2	C_3	C_4	C_1	C_2	C_3	C_4
**1A**	8	4	6.5	**1.5**	6.5	**1.5**	3	5
**2A**	2	7	5.5	3	4	5.5	**1**	8
**3A**	5	7	4	8	**1**	6	3	2
**Proximity**	4	8	2.5	7	5	2.5	**1**	6
**Average**	4.5	6.5	4.63	4.88	5.13	3.88	**2**	5.25

Table S16: Average ranks based on the disease-association analysis (cuarated databases) across the 8 datasets for the networks generated with ARACNE.

	**ARACNE (SE)**				**ARACNE (SN)**			
**Cat.**	C_1	C_2	C_3	C_4	C_1	C_2	C_3	C_4
**1A**	3	5	8	6	2	4	**1**	7
**2A**	5	**1**	2	3	7.5	6	7.5	4
**3A**	4.5	4.5	4.5	4.5	4.5	4.5	4.5	4.5
**Proximity**	6	5	4	**1**	7.5	3	7.5	2
**Average**	4.63	3.88	4.63	**3.63**	5.38	4.38	5.13	4.38

Table S17: Average ranks based on the disease-association analysis (curated databases) across the 8 datasets for the networks generated with MIC.

	**MIC (SE)**				**MIC (SN)**			
**Cat.**	C_1	C_2	C_3	C_4	C_1	C_2	C_3	C_4
**1A**	6	2	4	3	8	1	7	5
**2A**	7	3	8	**1.5**	5.5	**1.5**	5.5	4
**3A**	4	**1**	8	2	6	5	7	3
**Proximity**	5.5	**1**	3	4	7	2	8	5.5
**Average**	5.63	**1.75**	5.75	2.63	6.63	2.38	6.88	4.38

6 Case study: prostate cancer dataset

In this section we report the additional results from the analysis performed using the prostate dataset (Singh *et al.*, 2002) as a case study. In particular we show: 1) the overlap of enriched terms between co-prediction and co-expression networks, 2) the overlap between GO terms associated to the hubs of the networks generated with different methods and FuNeL and 3) the average percentages of alteration for key nodes of both co-prediction and co-expression networks in an independent dataset.

6.1 Overlap of networks enriched terms

We performed an analysis on the enriched terms of each network to highlight the complementary nature of the co-prediction and the co-expression paradigm. We generated heatmaps showing the unique terms associated only to co-prediction or co-expression networks. The main manuscript includes the comparison between FuNeL and Pearson networks, in here we report the analysis performed considering ARACNE (Figure S2) and MIC (Figure S3) networks. For the sake of readability we filtered out the generic GO terms (with depth < 9 in the GO hierarchical structure).

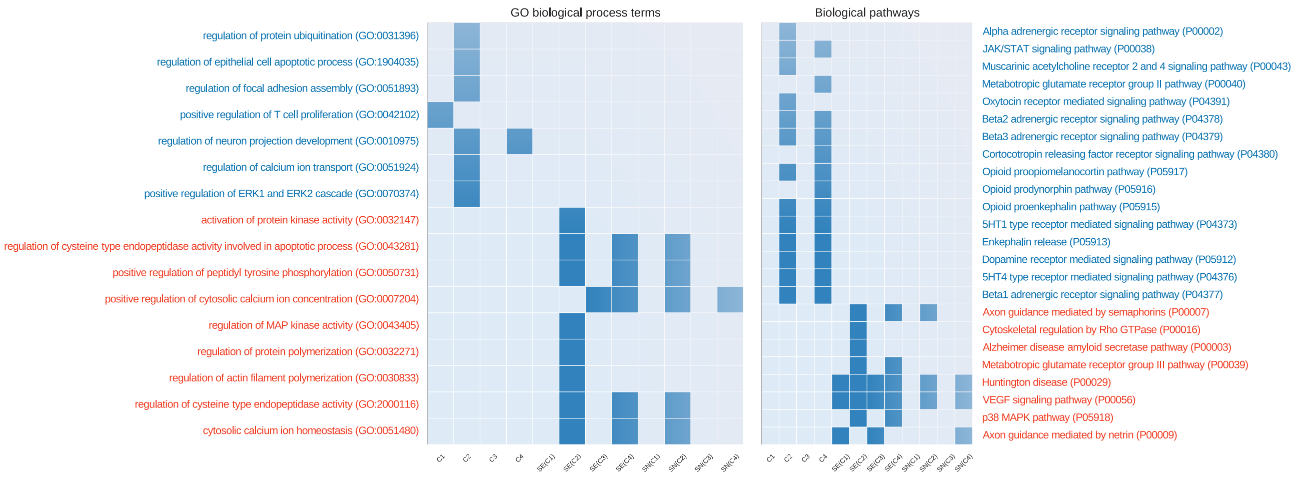

Figure S2: Number of non-common enriched GO terms (biological process) for each network configuration (generated from the prostate cancer dataset). On the x-axis we show the 12 investigated networks. On the y-axis we show the names of enriched terms unique to co-prediction or ARACNE co-expression networks. Red terms are associated with co-expression networks, blue with co-prediction. Empty columns indicate networks with no unique terms.

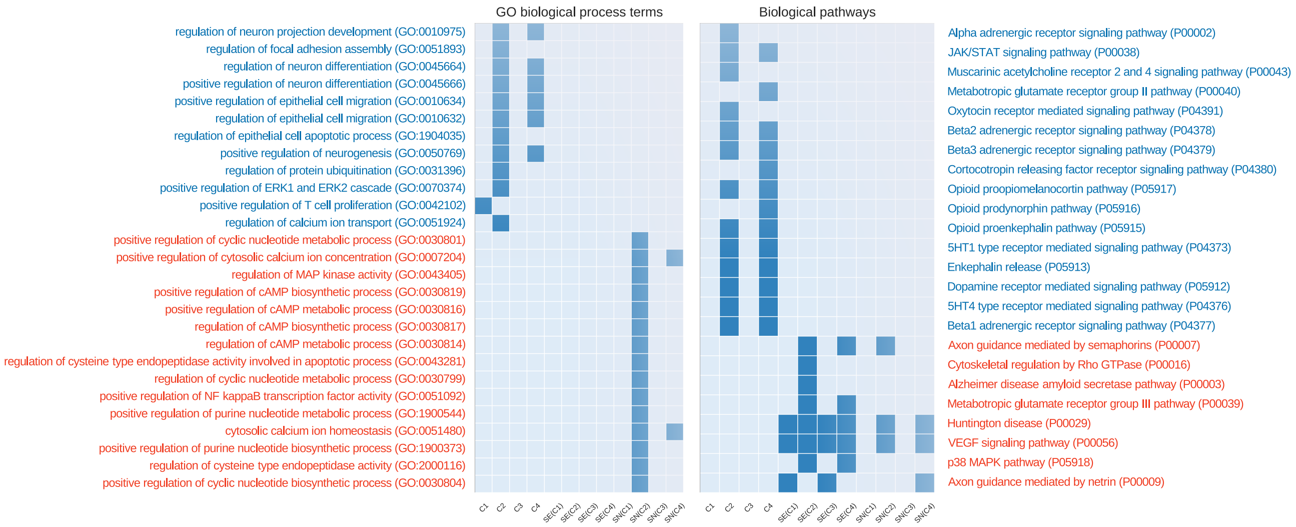

Figure S3: Number of non-common enriched GO terms (biological process) for each network configuration (generated from the prostate cancer dataset). On the x-axis we show the 12 investigated networks. On the y-axis we show the names of enriched terms unique to co-prediction or MIC co-expression networks. Red terms are associated with co-expression networks, blue with co-prediction. Empty columns indicate networks with no unique terms.

When comparing ARACNE and FuNeL, we found 16 unique pathways for co-prediction networks and 8 for co-expression. In terms of unique GO terms, the overlap was more balanced, 7 for co-prediction networks and 9 for co-expression networks. C_2 and C_4, generated without feature selection, had the largest number of unique pathways, while $SE(C_2)$ had the highest number of terms for ARACNE. The comparison of FuNeL with MIC generated many empty columns (Figure S3) for the GO terms because several networks resulted having no unique enriched terms. All the 15 unique GO terms related to MIC were associated to $SN(C_2)$ (and with $SN(C_4)$ in two cases), conversely FuNeL had more networks sharing the 12 unique terms. Finally, as noticed for in the ARACNE comparison, FuNeL networks are more enriched in biological pathways: 16 against 8 unique terms for MIC co-expression.

6.2 Overlap of hubs related terms

We also analysed the gene associated to the hubs of each network in order to compare the biological knowledge associated to them. A node v was considered to be a hub if its degree was at least one standard deviation above the mean network degree, that is if:

$$d(v) > \mu_d + \sigma_d \tag{4}$$

where $d(v)$ is a degree of the node v, and μ_d and σ_d are the mean and standard deviation of a network node degree distribution.

To compare the networks, we used the 10 most frequent GO terms (biological processes) shared among each network's hubs. Figure S4, S5 and S6 show the terms-overlap analysis between FuNeL networks and Pearson, ARACNE and MIC respectively. To make this analysis more specific we have discarded the most generic / most common terms (which could be be associated with many genes), we considered only the GO terms situated at level 10 of the GO hierarchy or lower. Blue terms were found only in co-prediction networks, red terms were found only in co-expression networks, and green terms were found in both. In Table S18 we summarise the number of unique and common terms shared between networks created with different approaches. This analysis further highlights the complementary nature of co-prediction and co-expression approach, the terms that are paradigm-specif always outnumber the common ones.

Table S18: Unique and common terms from networks' hubs

**Terms**	**Pearson**	**ARACNE**	**MIC**
**Co-prediction**	16	18	16
**Co-expression**	19	20	19
**Common**	11	9	11

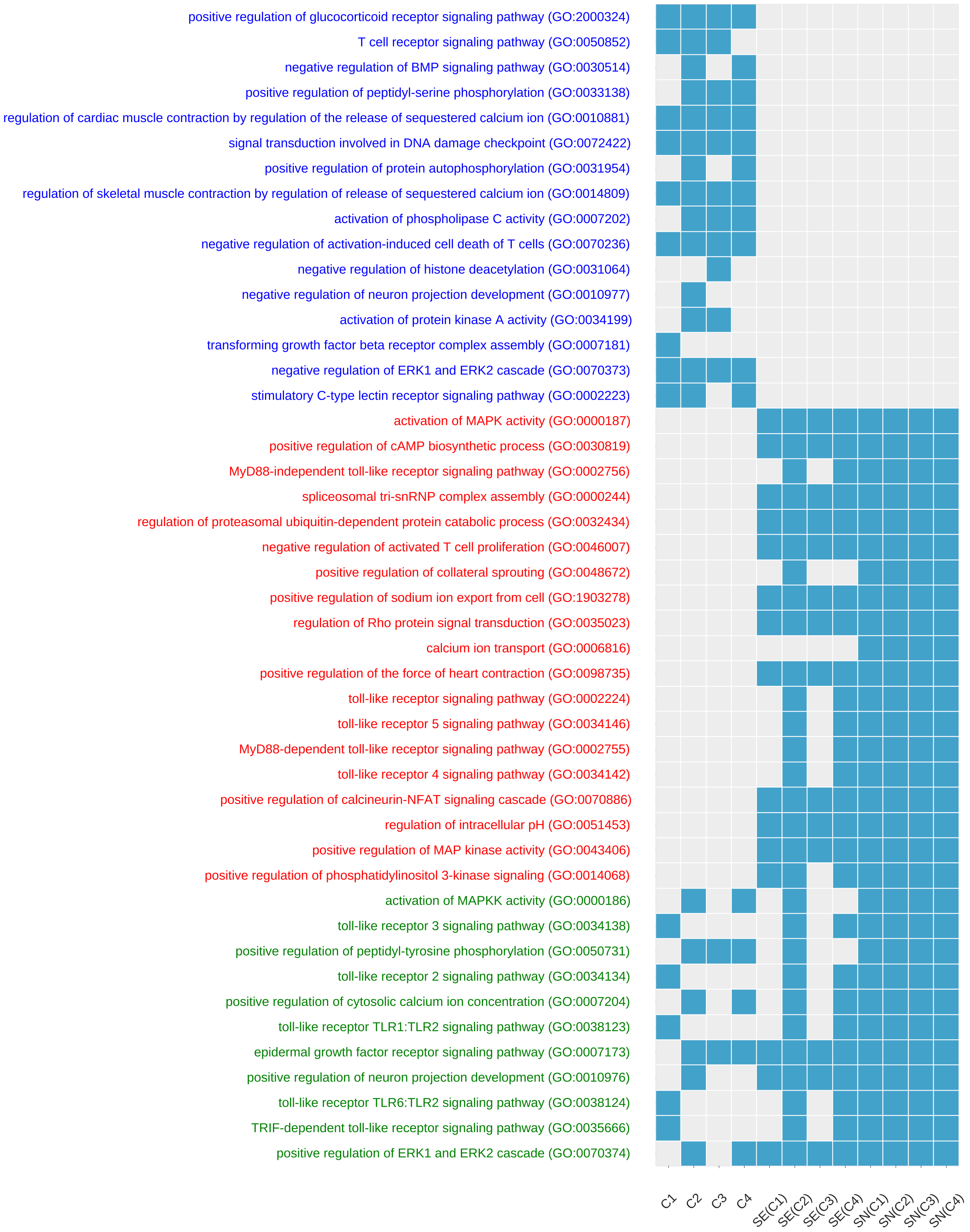

Figure S4: Top 10 most frequent biological processes from Gene Ontology found in the network hubs when comparing FuNeL and Pearson co-expression networks.

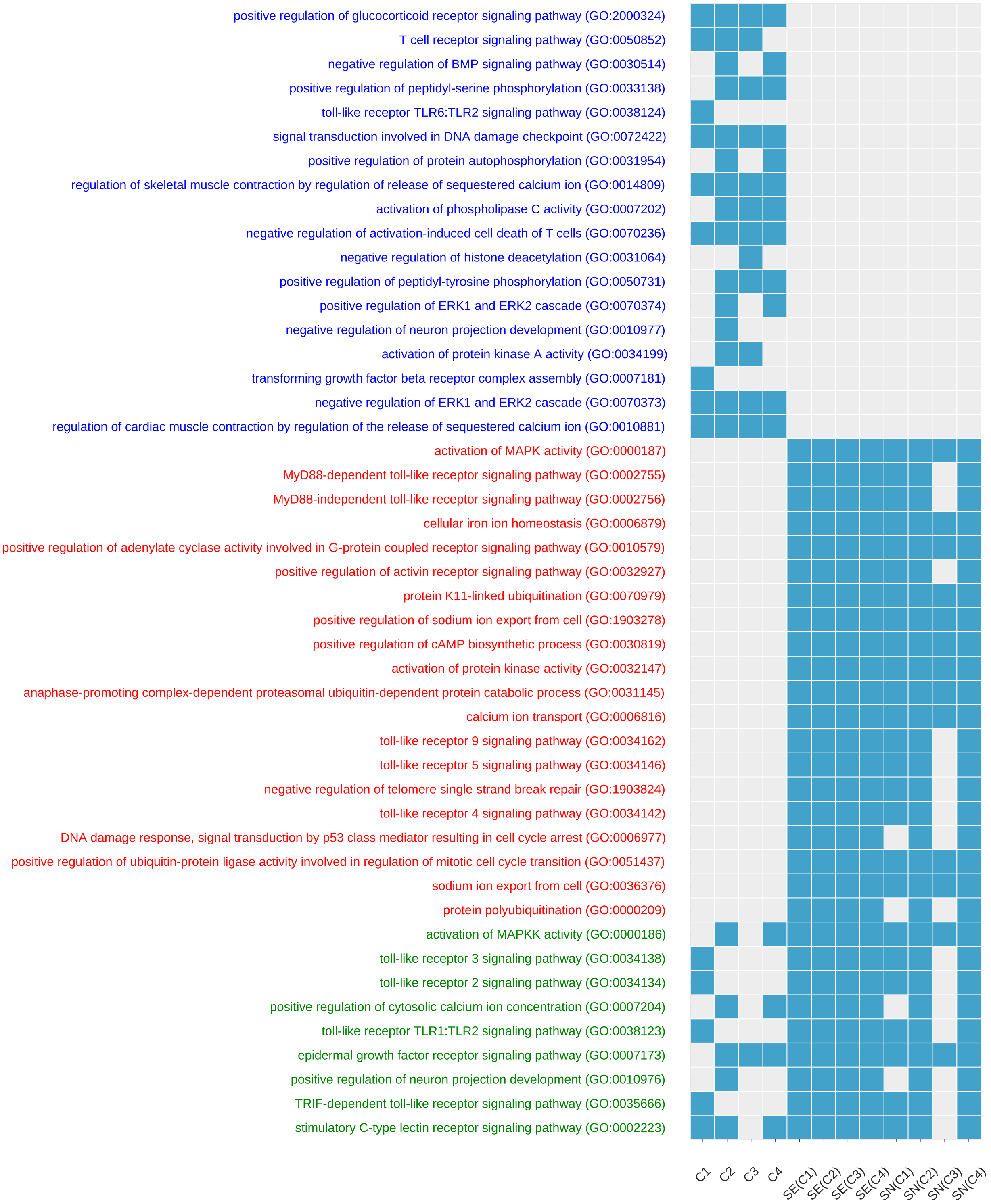

Figure S5: Top 10 most frequent biological processes from Gene Ontology found in the network hubs when comparing FuNeL and ARACNE co-expression networks.

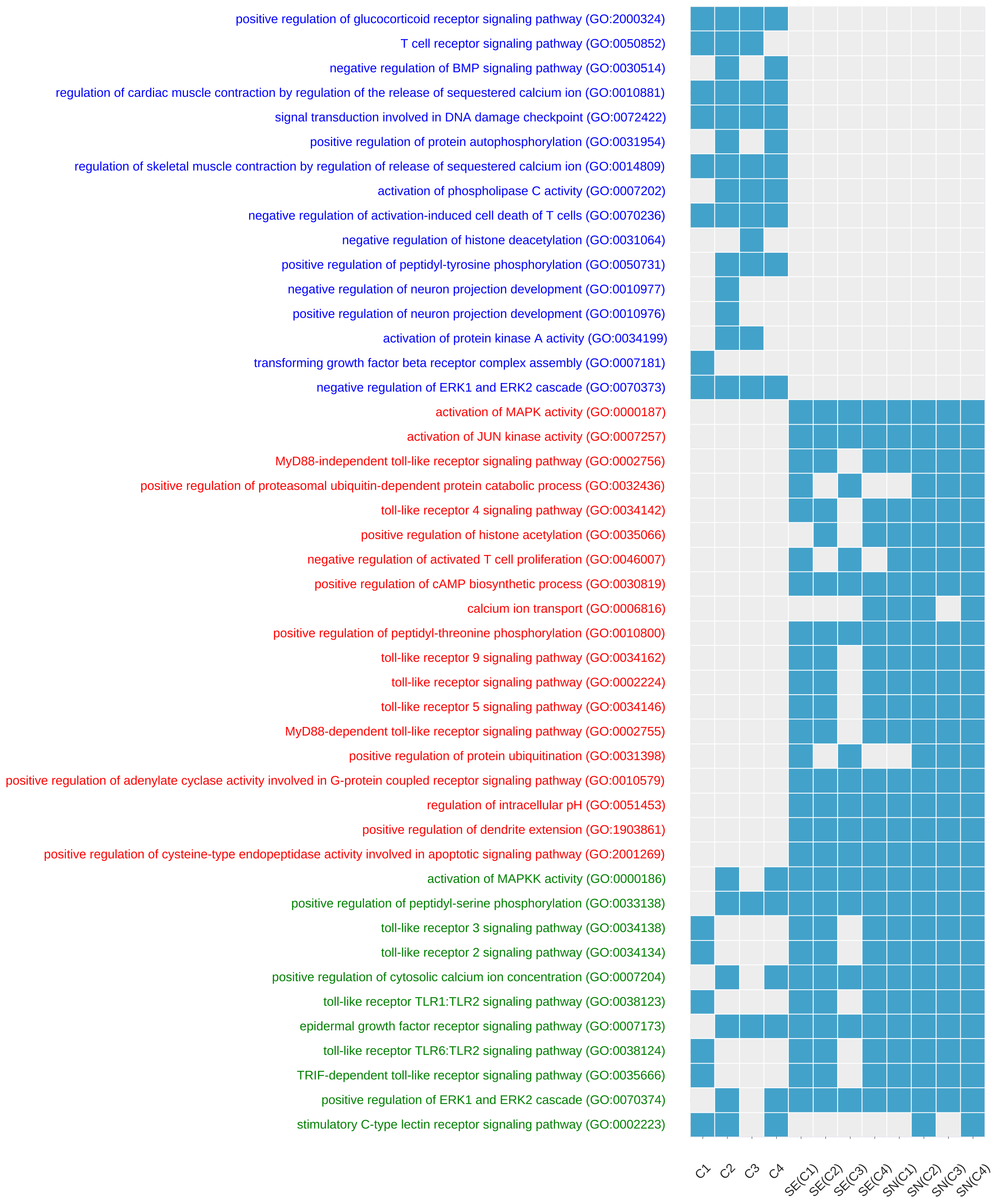

Figure S6: Top 10 most frequent biological processes from Gene Ontology found in the network hubs when comparing FuNeL and MIC co-expression networks.

6.3 Validation on independent dataset

In this section we report additional informations about the analysis performed using data from the independent prostate cancer study (Taylor *et al.*, 2010) available in the cBioPortal for Cancer Genomics (Cerami *et al.*, 2012). In particular we report the full list of alterations for the topologically important genes analysed in the main article. The Figure S7–S14 show the percentage of altered tumour samples for top 10 hubs (nodes with highest degree) and top 10 central nodes (with highest betweenness centrality) in the best performing networks according to the gene-disease association analysis (using the information from the curated databases). The selected networks are C_2 for FuNeL, $SN(C_3)$ for Pearson, $SE(C_4)$ for ARACNE and $SE(C_2)$ for MIC. For all of them we report the alterations for both hubs and central nodes.

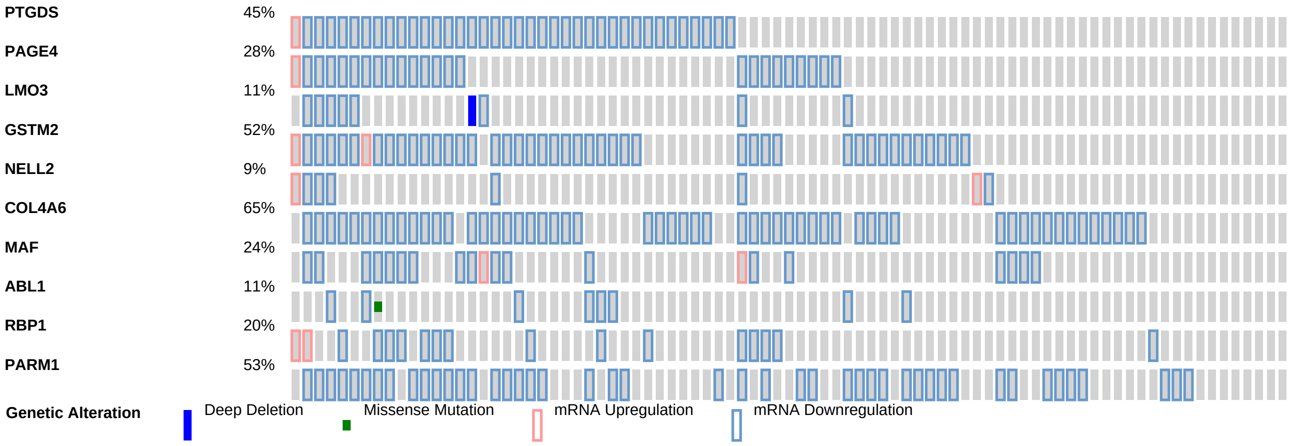

Figure S7: Percentage of alterations in tumour samples from an independent cancer genomic study. Genes with **highest degree** (hubs) in C_2 network are shown.

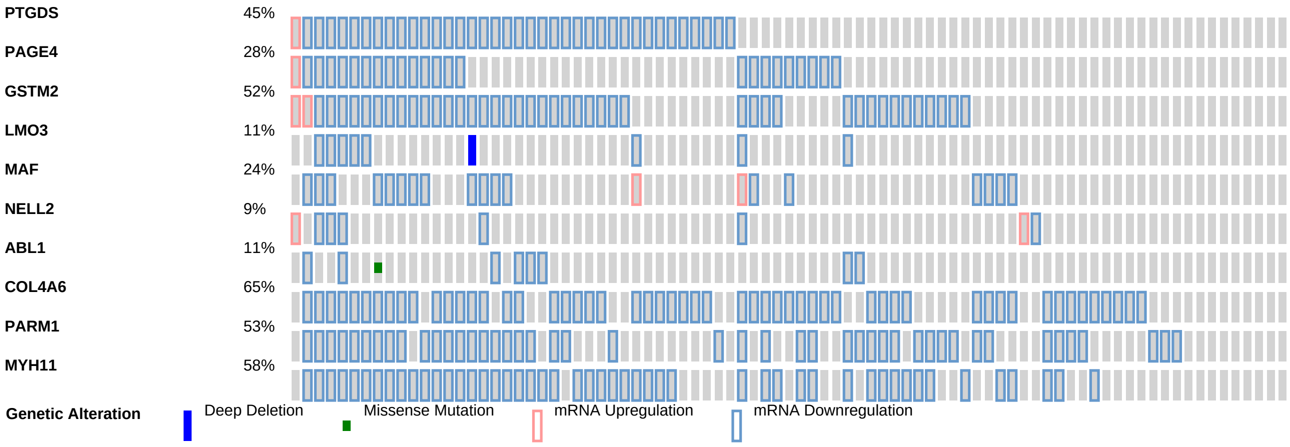

Figure S8: Percentage of alterations in tumour samples from an independent cancer genomic study. Genes with **highest betweenness centrality** (central nodes) in C_2 network are shown.

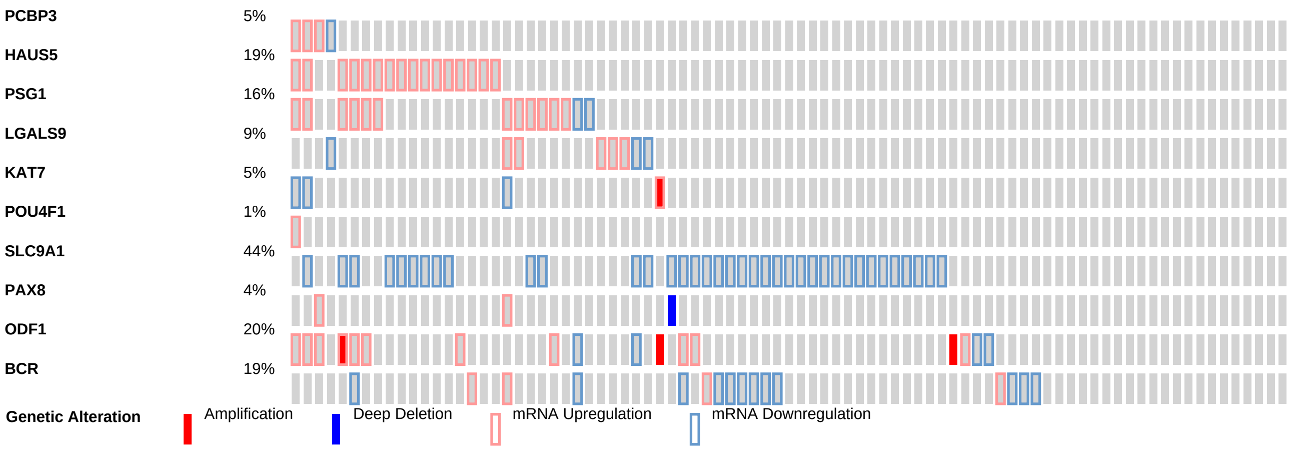

Figure S9: Percentage of alterations in tumour samples from an independent cancer genomic study. Genes with **highest degree** (hubs) in Pearson $SN(C_3)$ network are shown.

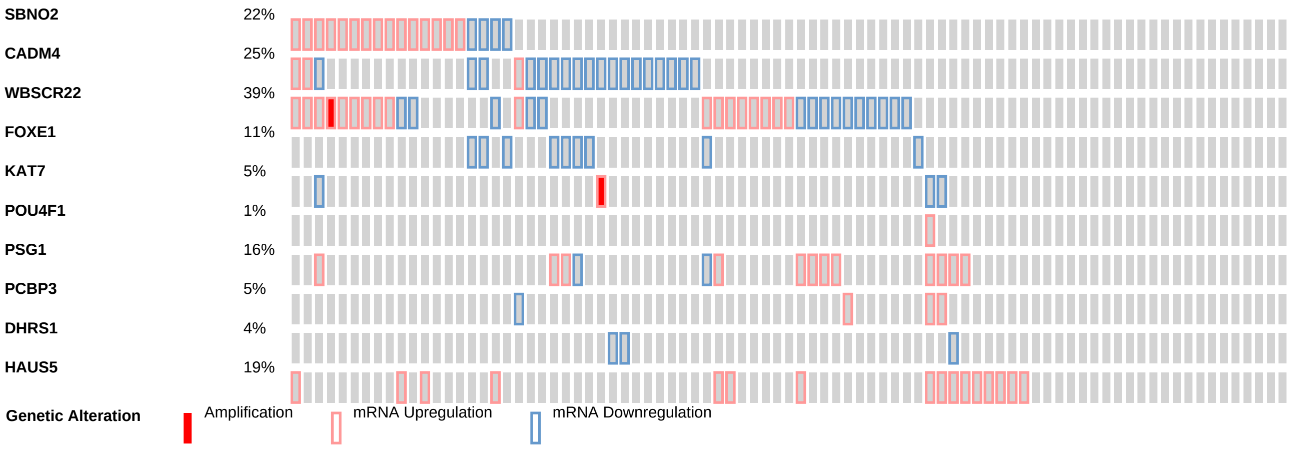

Figure S10: Percentage of alterations in tumour samples from an independent cancer genomic study. Genes with **highest betweenness centrality** (central nodes) in Pearson $SN(C_3)$ network are shown.

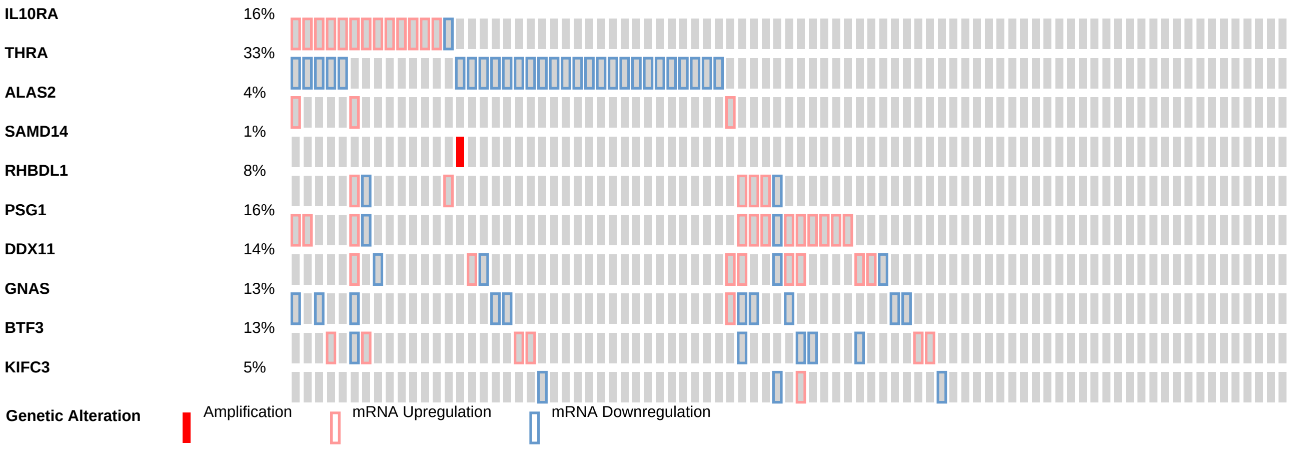

Figure S11: Percentage of alterations in tumour samples from an independent cancer genomic study. Genes with **highest degree** (hubs) in ARACNE $SE(C_4)$ network are shown.

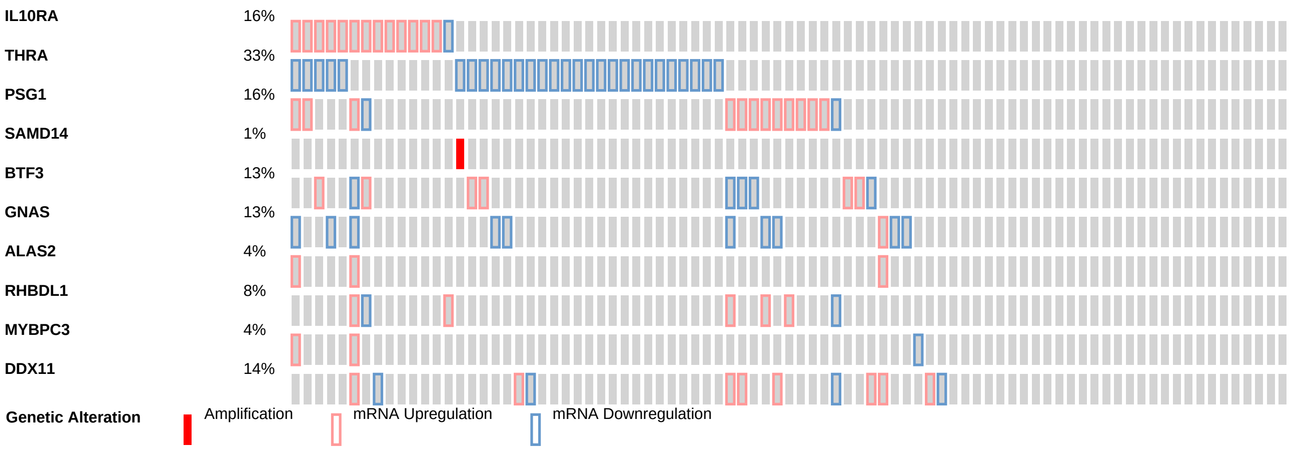

Figure S12: Percentage of alterations in tumour samples from an independent cancer genomic study. Genes with **highest betweenness centrality** (central nodes) in ARACNE $SE(C_4)$ network are shown.

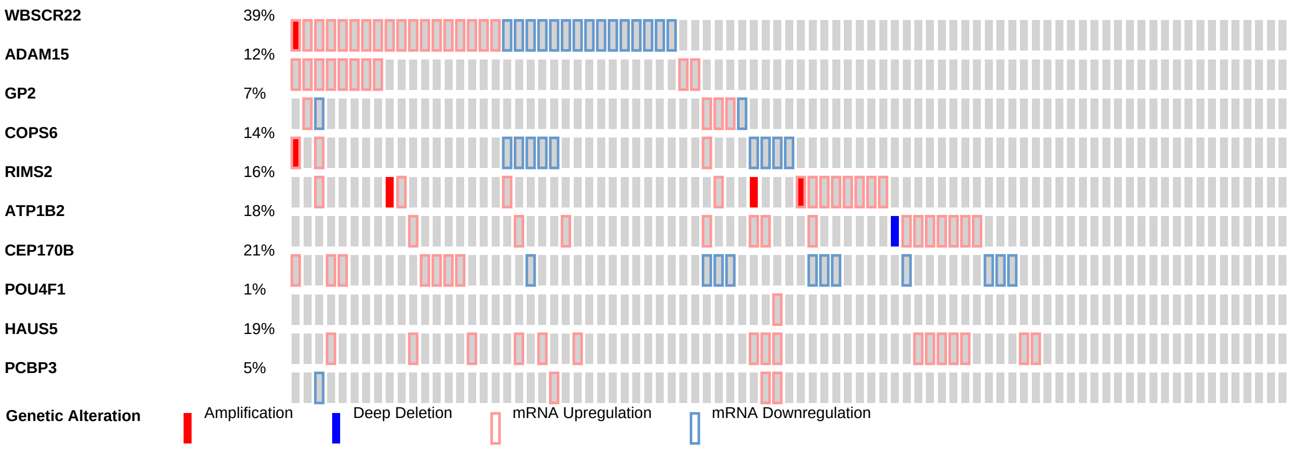

Figure S13: Percentage of alterations in tumour samples from an independent cancer genomic study. Genes with **highest degree** (hubs) in MIC $SE(C_2)$ network are shown.

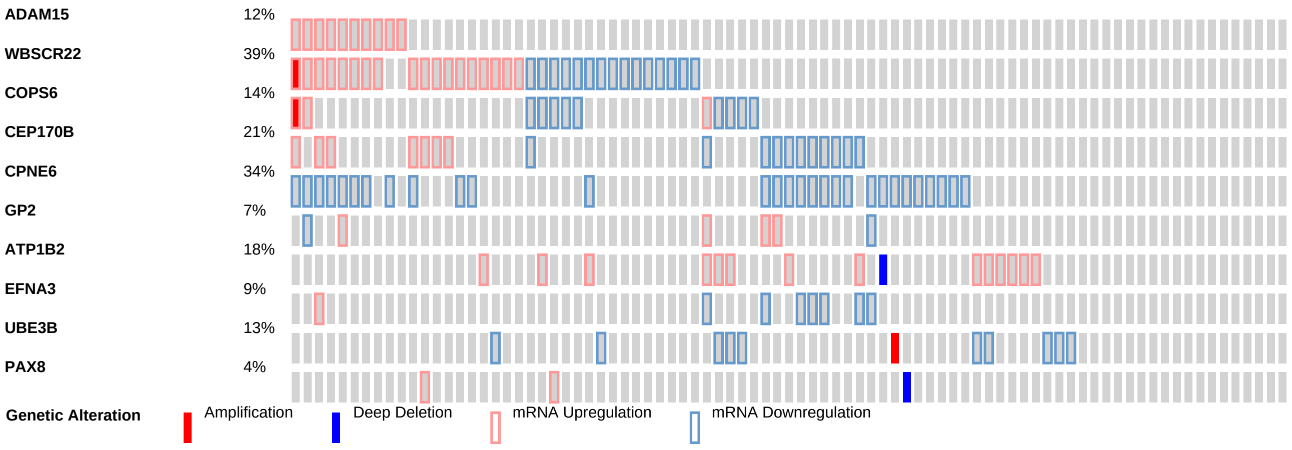

Figure S14: Percentage of alterations in tumour samples from an independent cancer genomic study. Genes with **highest betweenness centrality** (central nodes) in MIC $SE(C_2)$ network are shown.

7 Visualisation of the co-prediction and co-expression networks

In this section we include the layouts of co-prediction and co-expression networks for three of the datasets used in the main article: *Prostate* (used in the case study) and *Lung-Michigan* (small networks). Only a few examples are shown, for which the differences in the topology of the networks generated with the two approaches is the most visible. The networks were visualised using the *Organic Layout* in Cytoscape (Shannon *et al.*, 2003).

Prostate: FuNeL – C_1

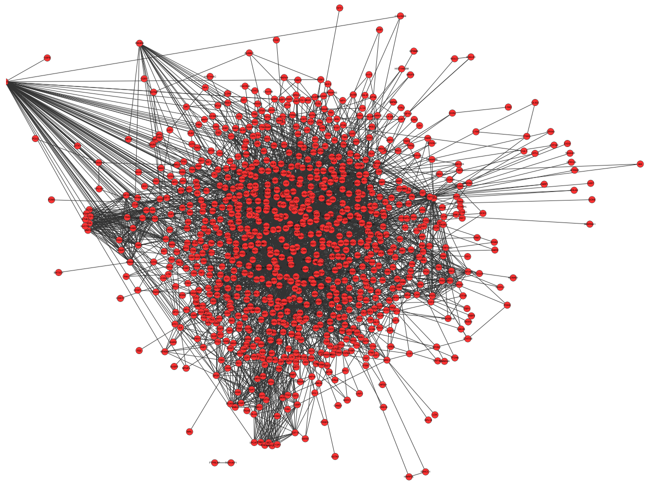

Prostate: FuNeL – C_3

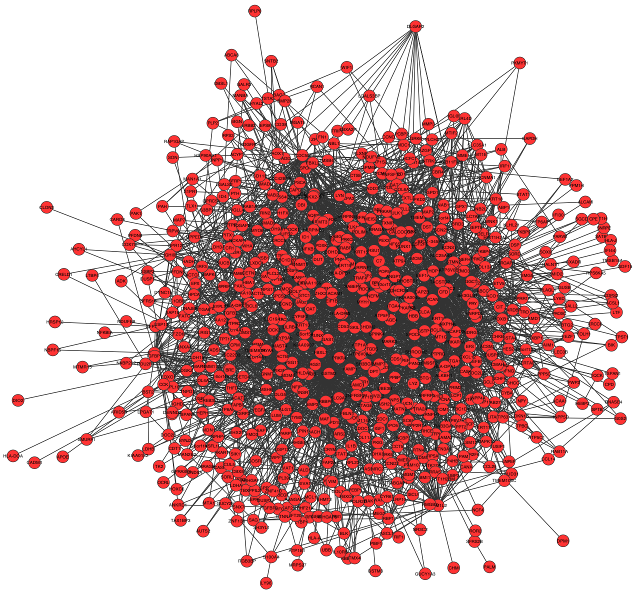

Prostate: ARACNE – $SN(C_1)$

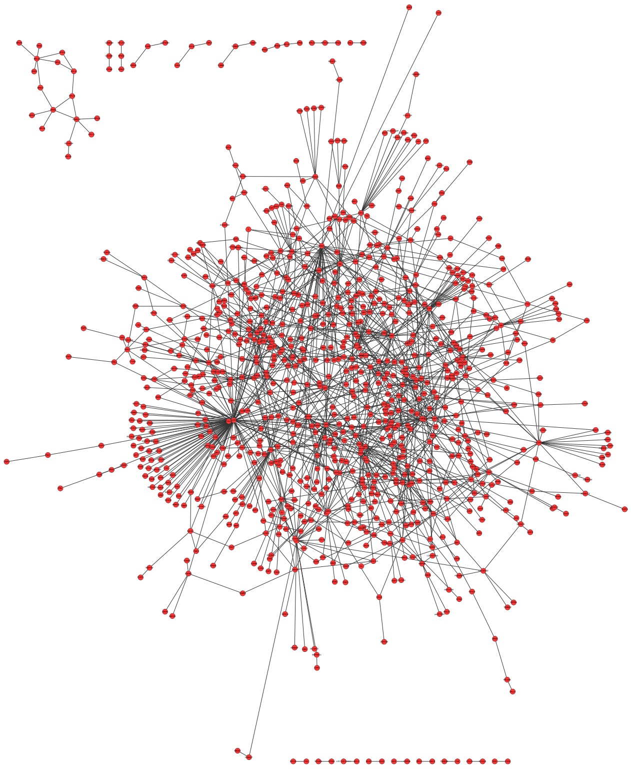

Prostate: ARACNE – $SE(C_3)$

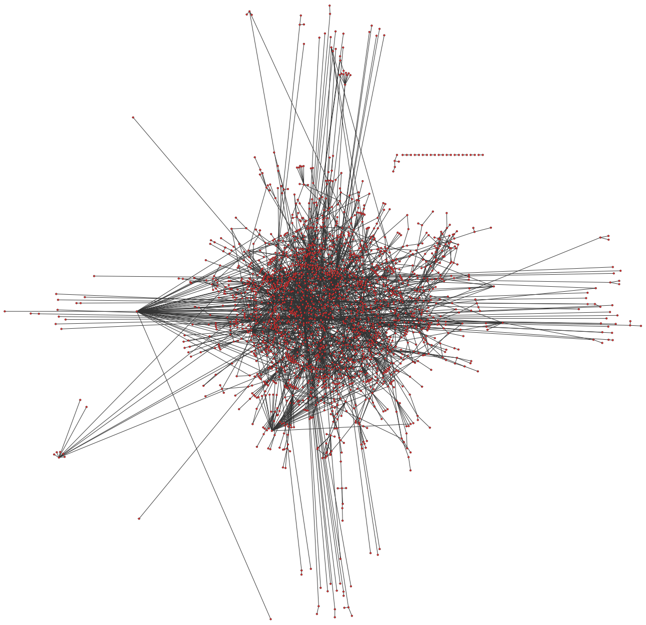

Prostate: Pearson – $SN(C_1)$

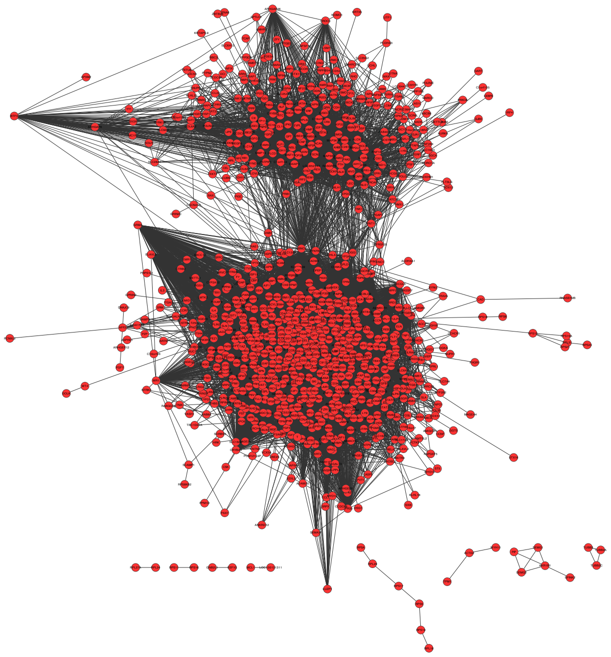

Prostate: Pearson – $SE(C_3)$

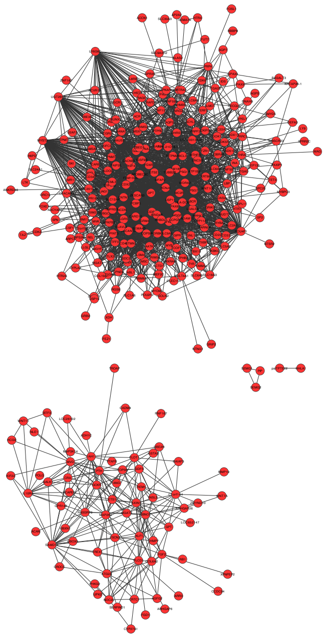

Prostate: MIC – $SN(C_1)$

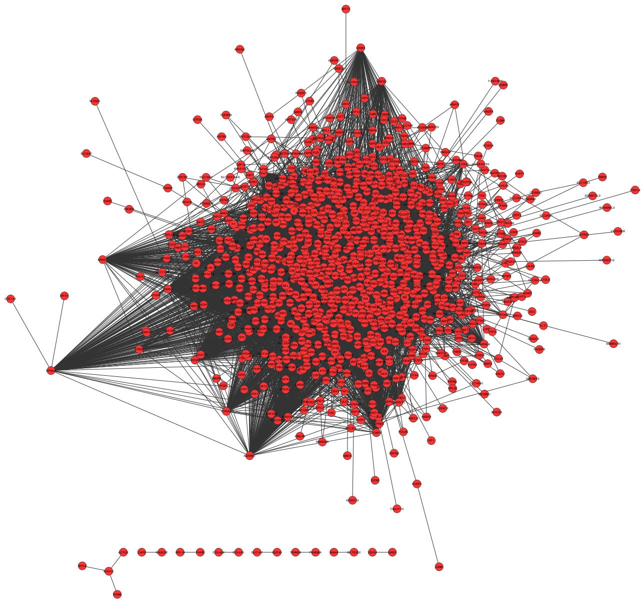

Prostate: MIC – $SE(C_3)$

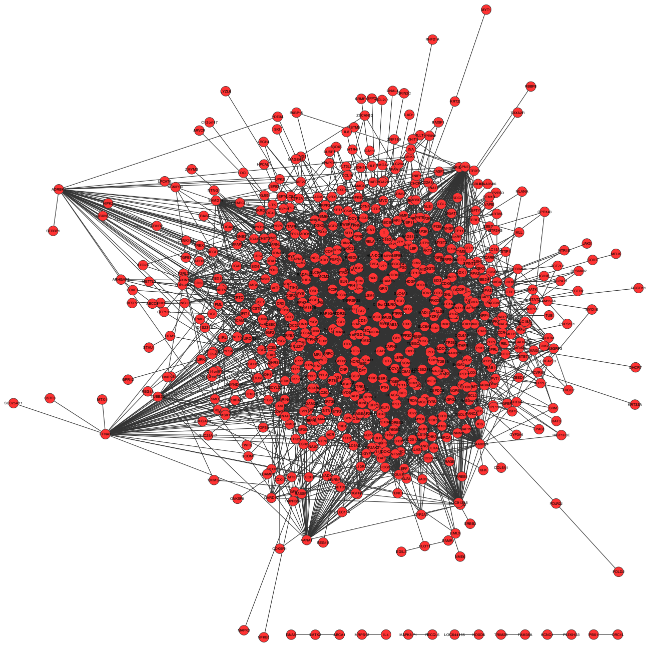

Lung-Michigan: FuNeL $- C_2$

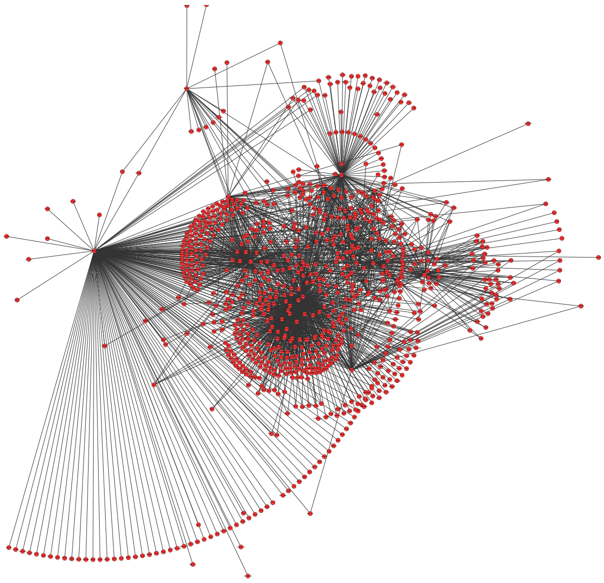

Lung-Michigan: FuNeL $- C_4$

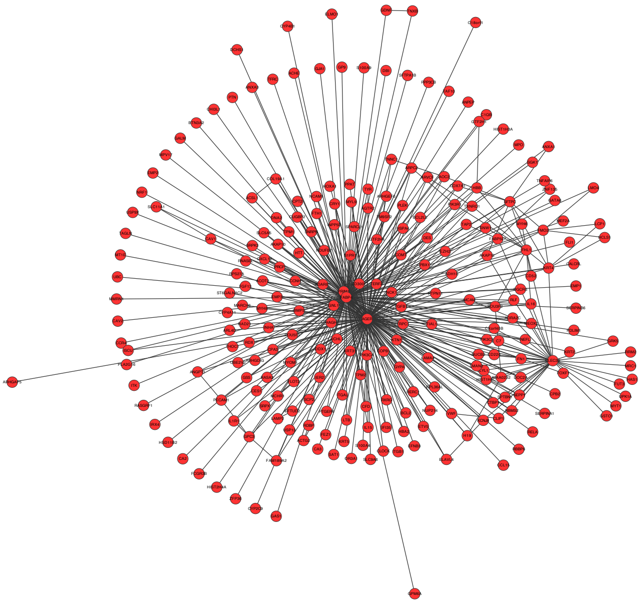

Lung-Michigan: ARACNE $- SE(C_2)$

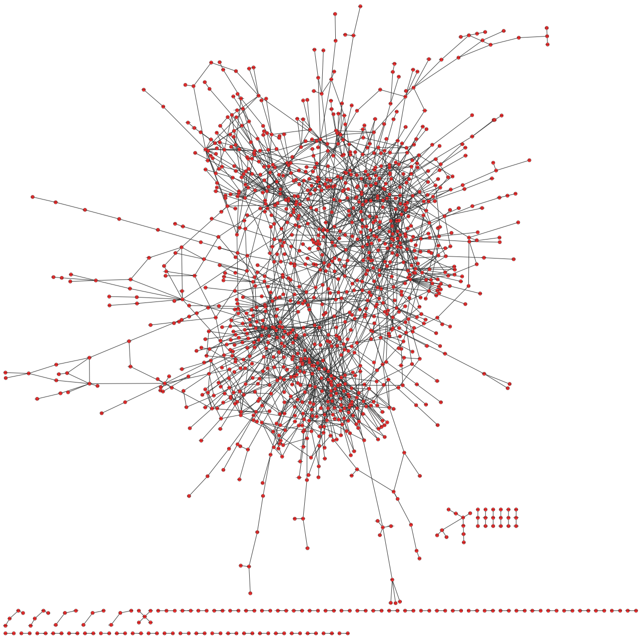

Lung-Michigan: ARACNE $- SN(C_4)$

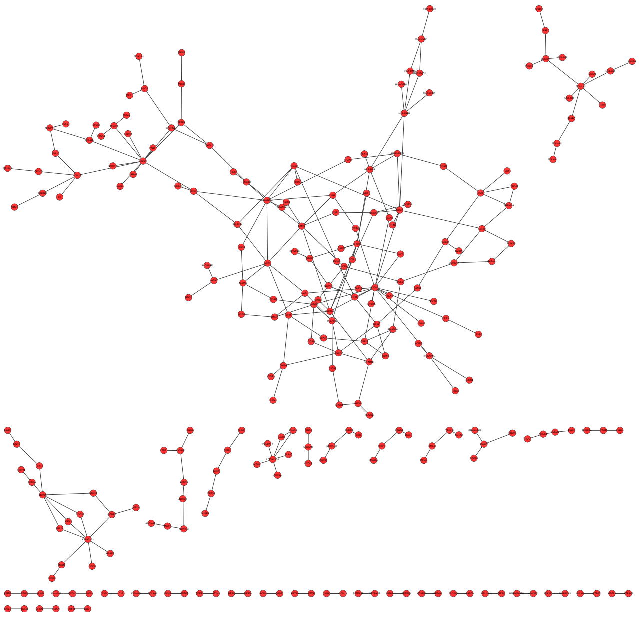

Lung-Michigan: Pearson $- SE(C_2)$

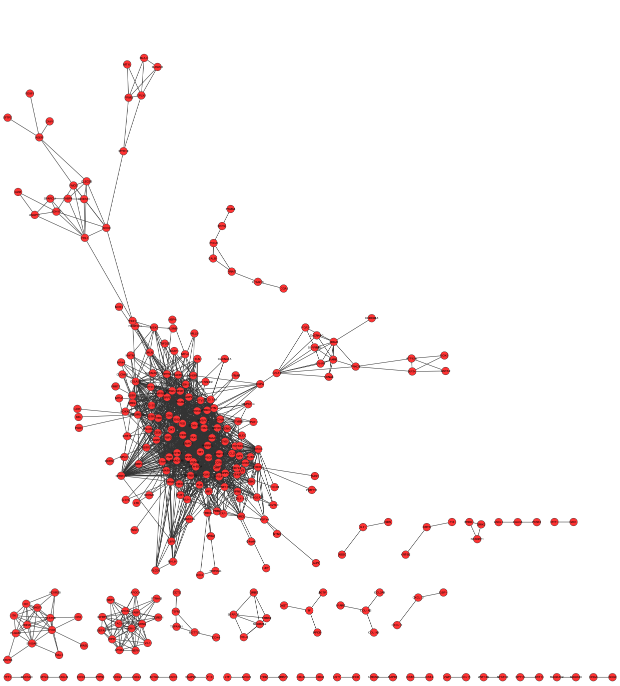

Lung-Michigan: Pearson $- SN(C_4)$

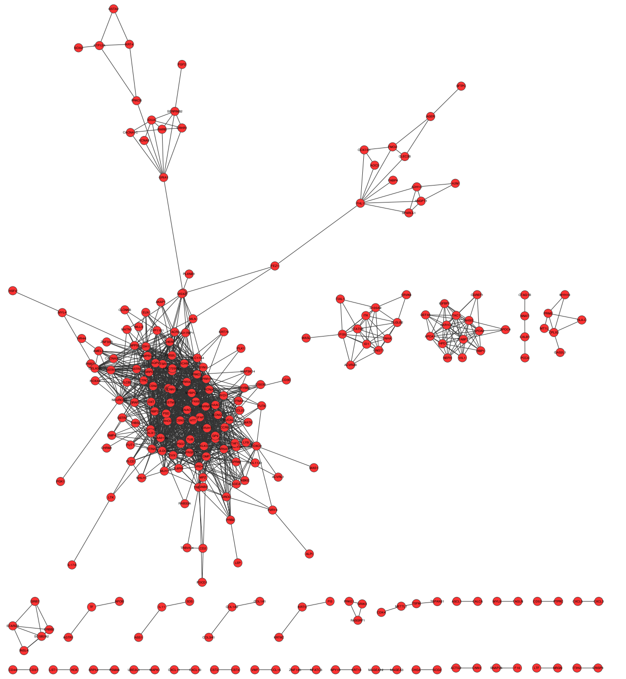

Lung-Michigan: MIC $- SE(C_2)$

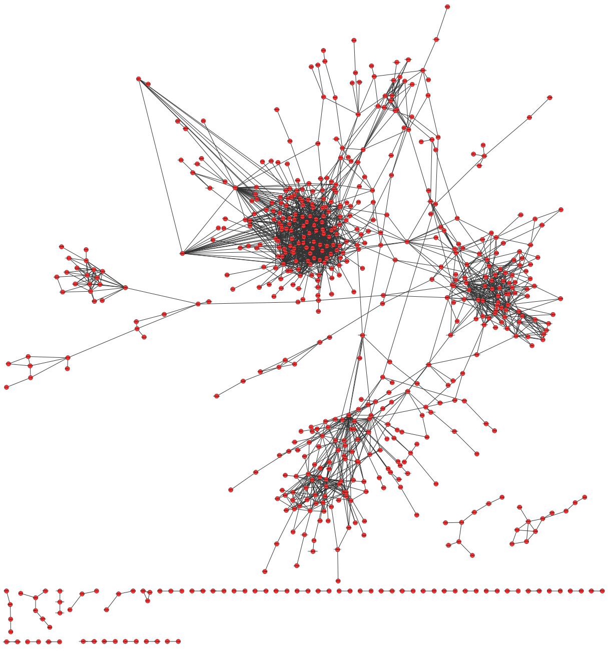

Lung-Michigan: MIC $- SN(C_4)$

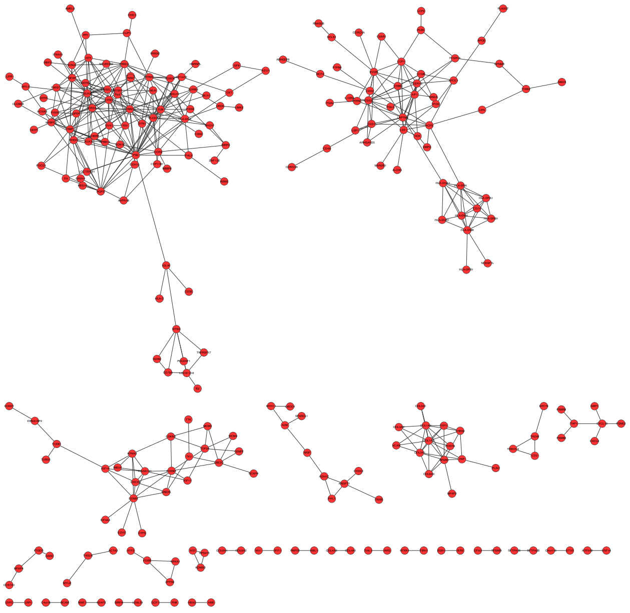

References

Cerami, E. et al (2012). The cbio cancer genomics portal: An open platform for exploring multidimensional cancer genomics data. *Cancer Discovery*, **2**(5), 401–404.

Chowdary, D. et al (2006). Prognostic gene expression signatures can be measured in tissues collected in RNAlater preservative. *The Journal of Molecular Diagnostics*, **8**(1), 31 – 39.

Davis, A.P. et al (2014). The comparative toxicogenomics database's 10th year anniversary: update 2015. *Nucleic Acids Research*.

Hamosh, A. et al (2002). Online mendelian inheritance in man (omim), a knowledgebase of human genes and genetic disorders. *Nucleic Acids Res*, pages 52–55.

Magrane, M. et al (2011). Uniprot knowledgebase: a hub of integrated protein data. *Database*, **2011**.

Orphanet (1997). Orphanet: an online database of rare diseases and orphan drugs. copyright, inserm 1997.

Rappaport, N. et al (2013). Malacards: an integrated compendium for diseases and their annotation. *Database*, **2013**.

Shannon, P. et al (2003). Cytoscape: a software environment for integrated models of biomolecular interaction networks. *Genome Research*, **13**(11), 2498–2504.

Singh, D. et al (2002). Gene expression correlates of clinical prostate cancer behavior. *Cancer Cell*, **1**(2), 203 – 209.

Taylor, B.S. et al (2010). Integrative genomic profiling of human prostate cancer. *Cancer Cell*, **18**(1), 11 – 22.
