# Supplementary material for: Functional networks inference from rule-based machine learning models
Source: BioData Min. 2016 Sep 5;9:28. doi: 10.1186/s13040-016-0106-4 (PMC5011349; doi:10.1186/s13040-016-0106-4)
Supplement: Supplementary file 4 — Enriched terms. Complete list of enriched terms for each dataset and network. (XZ 3041 kb) [file 13040_2016_106_MOESM4_ESM.xz › enriched_terms/co_prediction/hubs_heatmap_co_expression.pdf]

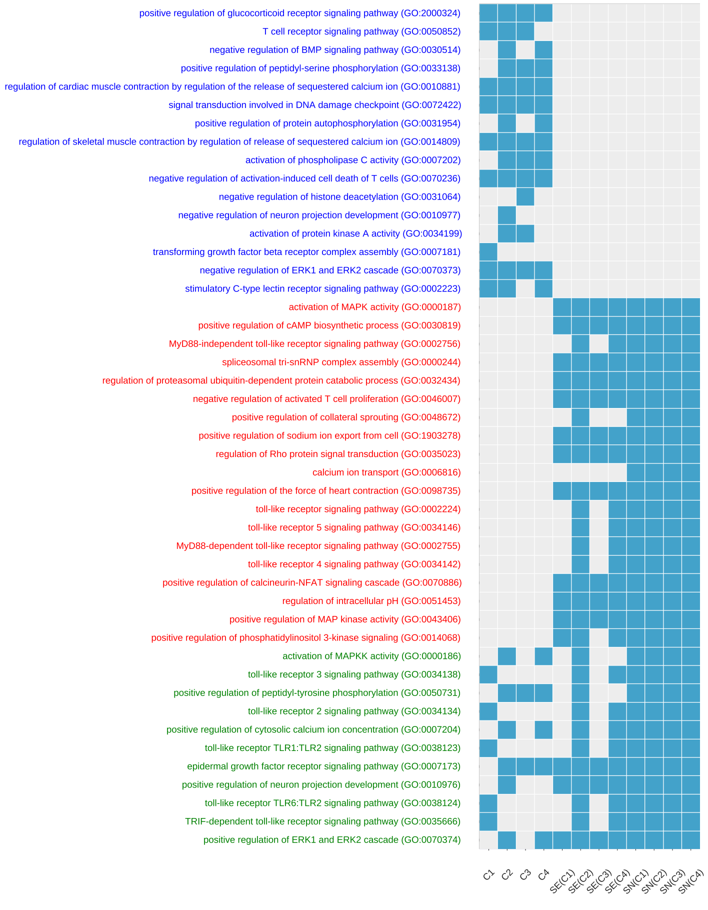
positive regulation of glucocorticoid receptor signaling pathway (GO:2000324)
T cell receptor signaling pathway (GO:0050852)
negative regulation of BMP signaling pathway (GO:0030514)
positive regulation of peptidyl-serine phosphorylation (GO:0033138)
regulation of cardiac muscle contraction by regulation of the release of sequestered calcium ion (GO:0010881)
signal transduction involved in DNA damage checkpoint (GO:0072422)
positive regulation of protein autophosphorylation (GO:0031954)
regulation of skeletal muscle contraction by regulation of release of sequestered calcium ion (GO:0014809)
activation of phospholipase C activity (GO:0007202)
negative regulation of activation-induced cell death of T cells (GO:0070236)
negative regulation of histone deacetylation (GO:0031064)
negative regulation of neuron projection development (GO:0010977)
activation of protein kinase A activity (GO:0034199)
transforming growth factor beta receptor complex assembly (GO:0007181)
negative regulation of ERK1 and ERK2 cascade (GO:0070373)
stimulatory C-type lectin receptor signaling pathway (GO:0002223)
activation of MAPK activity (GO:0000187)
positive regulation of cAMP biosynthetic process (GO:0030819)
MyD88-independent toll-like receptor signaling pathway (GO:0002756)
spliceosomal tri-snRNP complex assembly (GO:0000244)
regulation of proteasomal ubiquitin-dependent protein catabolic process (GO:0032434)
negative regulation of activated T cell proliferation (GO:0046007)
positive regulation of collateral sprouting (GO:0048672)
positive regulation of sodium ion export from cell (GO:1903278)
regulation of Rho protein signal transduction (GO:0035023)
calcium ion transport (GO:0006816)
positive regulation of the force of heart contraction (GO:0098735)
toll-like receptor signaling pathway (GO:0002224)
toll-like receptor 5 signaling pathway (GO:0034146)
MyD88-dependent toll-like receptor signaling pathway (GO:0002755)
toll-like receptor 4 signaling pathway (GO:0034142)
positive regulation of calcineurin-NFAT signaling cascade (GO:0070886)
regulation of intracellular pH (GO:0051453)
positive regulation of MAP kinase activity (GO:0043406)
positive regulation of phosphatidylinositol 3-kinase signaling (GO:0014068)
activation of MAPKK activity (GO:0000186)
toll-like receptor 3 signaling pathway (GO:0034138)
positive regulation of peptidyl-tyrosine phosphorylation (GO:0050731)
toll-like receptor 2 signaling pathway (GO:0034134)
positive regulation of cytosolic calcium ion concentration (GO:0007204)
toll-like receptor TLR1:TLR2 signaling pathway (GO:0038123)
epidermal growth factor receptor signaling pathway (GO:0007173)
positive regulation of neuron projection development (GO:0010976)
toll-like receptor TLR6:TLR2 signaling pathway (GO:0038124)
TRIF-dependent toll-like receptor signaling pathway (GO:0035666)
positive regulation of ERK1 and ERK2 cascade (GO:0070374)
C1
C2
C3
C4
SE(C1)
SE(C2)
SE(C3)
SE(C4)
SN(C1)
SN(C2)
SN(C3)
SN(C4)
