# Supplementary material for: Functional networks inference from rule-based machine learning models
Source: BioData Min. 2016 Sep 5;9:28. doi: 10.1186/s13040-016-0106-4 (PMC5011349; doi:10.1186/s13040-016-0106-4)
Supplement: Supplementary file 4 — Enriched terms. Complete list of enriched terms for each dataset and network. (XZ 3041 kb) [file 13040_2016_106_MOESM4_ESM.xz › enriched_terms/co_prediction/heatmaps_mic.pdf]

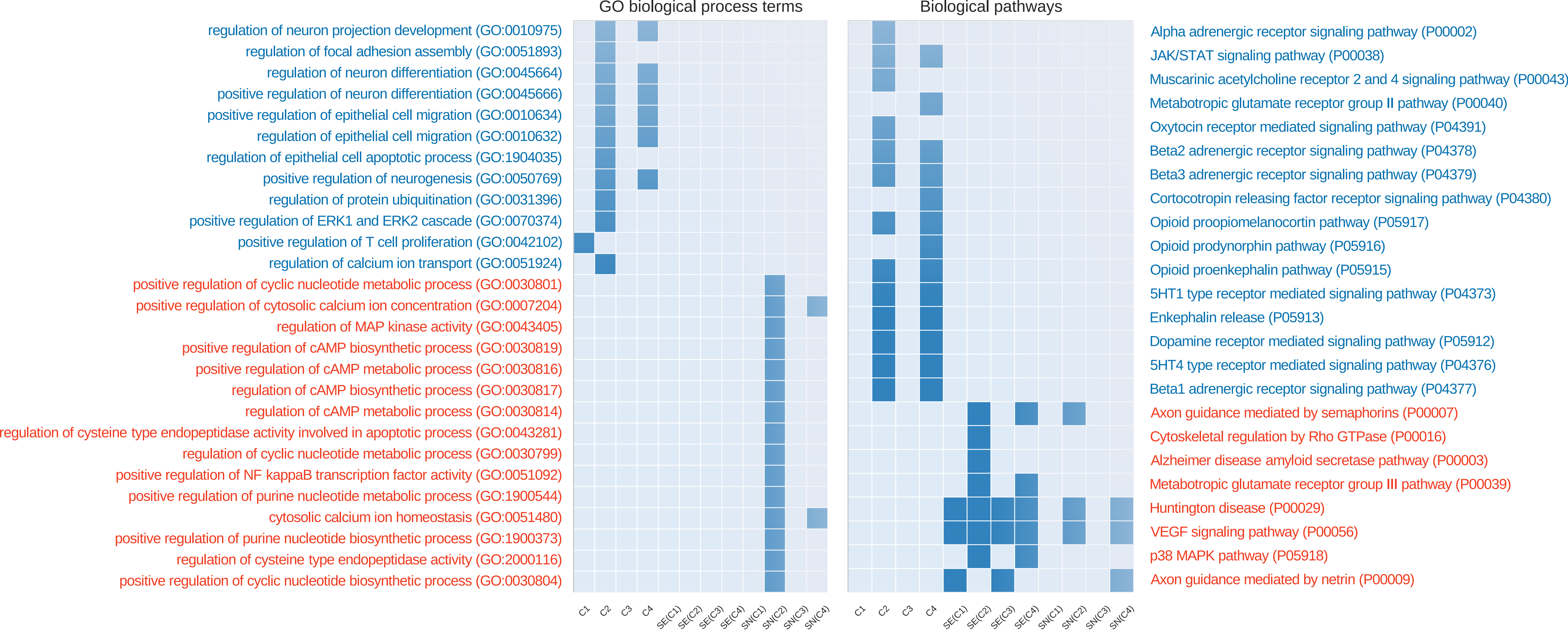
GO biological process terms
Biological pathways
regulation of neuron projection development (GO:0010975)
regulation of focal adhesion assembly (GO:0051893)
regulation of neuron differentiation (GO:0045664)
positive regulation of neuron differentiation (GO:0045666)
positive regulation of epithelial cell migration (GO:0010634)
regulation of epithelial cell migration (GO:0010632)
regulation of epithelial cell apoptotic process (GO:1904035)
positive regulation of neurogenesis (GO:0050769)
regulation of protein ubiquitination (GO:0031396)
positive regulation of ERK1 and ERK2 cascade (GO:0070374)
positive regulation of T cell proliferation (GO:0042102)
regulation of calcium ion transport (GO:0051924)
positive regulation of cyclic nucleotide metabolic process (GO:0030801)
positive regulation of cytosolic calcium ion concentration (GO:0007204)
regulation of MAP kinase activity (GO:0043405)
positive regulation of cAMP biosynthetic process (GO:0030819)
positive regulation of cAMP metabolic process (GO:0030816)
regulation of cAMP biosynthetic process (GO:0030817)
regulation of cAMP metabolic process (GO:0030814)
regulation of cysteine type endopeptidase activity involved in apoptotic process (GO:0043281)
regulation of cyclic nucleotide metabolic process (GO:0030799)
positive regulation of NF kappaB transcription factor activity (GO:0051092)
positive regulation of purine nucleotide metabolic process (GO:1900544)
cytosolic calcium ion homeostasis (GO:0051480)
positive regulation of purine nucleotide biosynthetic process (GO:1900373)
regulation of cysteine type endopeptidase activity (GO:2000116)
positive regulation of cyclic nucleotide biosynthetic process (GO:0030804)
C1 C2 C3 C4 SE(C1) SE(C2) SE(C3) SE(C4) SN(C1) SN(C2) SN(C3) SN(C4)
C1 C2 C3 C4 SE(C1) SE(C2) SE(C3) SE(C4) SN(C1) SN(C2) SN(C3) SN(C4)
Alpha adrenergic receptor signaling pathway (P00002)
JAK/STAT signaling pathway (P00038)
Muscarinic acetylcholine receptor 2 and 4 signaling pathway (P00043)
Metabotropic glutamate receptor group II pathway (P00040)
Oxytocin receptor mediated signaling pathway (P04391)
Beta2 adrenergic receptor signaling pathway (P04378)
Beta3 adrenergic receptor signaling pathway (P04379)
Cortocotropin releasing factor receptor signaling pathway (P04380)
Opioid proopiomelanocortin pathway (P05917)
Opioid prodynorphin pathway (P05916)
Opioid proenkephalin pathway (P05915)
5HT1 type receptor mediated signaling pathway (P04373)
Enkephalin release (P05913)
Dopamine receptor mediated signaling pathway (P05912)
5HT4 type receptor mediated signaling pathway (P04376)
Beta1 adrenergic receptor signaling pathway (P04377)
Axon guidance mediated by semaphorins (P00007)
Cytoskeletal regulation by Rho GTPase (P00016)
Alzheimer disease amyloid secretase pathway (P00003)
Metabotropic glutamate receptor group III pathway (P00039)
Huntington disease (P00029)
VEGF signaling pathway (P00056)
p38 MAPK pathway (P05918)
Axon guidance mediated by netrin (P00009)
