# Supplementary material for: Functional networks inference from rule-based machine learning models
Source: BioData Min. 2016 Sep 5;9:28. doi: 10.1186/s13040-016-0106-4 (PMC5011349; doi:10.1186/s13040-016-0106-4)
Supplement: Supplementary file 4 — Enriched terms. Complete list of enriched terms for each dataset and network. (XZ 3041 kb) [file 13040_2016_106_MOESM4_ESM.xz › enriched_terms/co_prediction/hubs_heatmap_aracne.pdf]

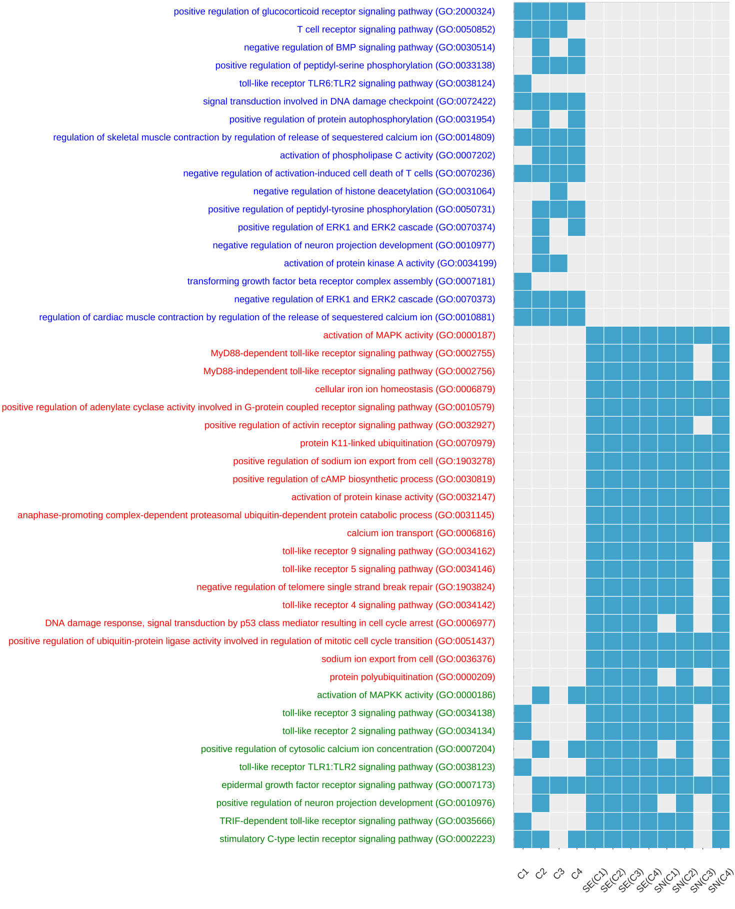
positive regulation of glucocorticoid receptor signaling pathway (GO:2000324)
T cell receptor signaling pathway (GO:0050852)
negative regulation of BMP signaling pathway (GO:0030514)
positive regulation of peptidyl-serine phosphorylation (GO:0033138)
toll-like receptor TLR6:TLR2 signaling pathway (GO:0038124)
signal transduction involved in DNA damage checkpoint (GO:0072422)
positive regulation of protein autophosphorylation (GO:0031954)
regulation of skeletal muscle contraction by regulation of release of sequestered calcium ion (GO:0014809)
activation of phospholipase C activity (GO:0007202)
negative regulation of activation-induced cell death of T cells (GO:0070236)
negative regulation of histone deacetylation (GO:0031064)
positive regulation of peptidyl-tyrosine phosphorylation (GO:0050731)
positive regulation of ERK1 and ERK2 cascade (GO:0070374)
negative regulation of neuron projection development (GO:0010977)
activation of protein kinase A activity (GO:0034199)
transforming growth factor beta receptor complex assembly (GO:0007181)
negative regulation of ERK1 and ERK2 cascade (GO:0070373)
regulation of cardiac muscle contraction by regulation of the release of sequestered calcium ion (GO:0010881)
activation of MAPK activity (GO:0000187)
MyD88-dependent toll-like receptor signaling pathway (GO:0002755)
MyD88-independent toll-like receptor signaling pathway (GO:0002756)
cellular iron ion homeostasis (GO:0006879)
positive regulation of adenylate cyclase activity involved in G-protein coupled receptor signaling pathway (GO:0010579)
positive regulation of activin receptor signaling pathway (GO:0032927)
protein K11-linked ubiquitination (GO:0070979)
positive regulation of sodium ion export from cell (GO:1903278)
positive regulation of cAMP biosynthetic process (GO:0030819)
activation of protein kinase activity (GO:0032147)
anaphase-promoting complex-dependent proteasomal ubiquitin-dependent protein catabolic process (GO:0031145)
calcium ion transport (GO:0006816)
toll-like receptor 9 signaling pathway (GO:0034162)
toll-like receptor 5 signaling pathway (GO:0034146)
negative regulation of telomere single strand break repair (GO:1903824)
toll-like receptor 4 signaling pathway (GO:0034142)
DNA damage response, signal transduction by p53 class mediator resulting in cell cycle arrest (GO:0006977)
positive regulation of ubiquitin-protein ligase activity involved in regulation of mitotic cell cycle transition (GO:0051437)
sodium ion export from cell (GO:0036376)
protein polyubiquitination (GO:0000209)
activation of MAPKK activity (GO:0000186)
toll-like receptor 3 signaling pathway (GO:0034138)
toll-like receptor 2 signaling pathway (GO:0034134)
positive regulation of cytosolic calcium ion concentration (GO:0007204)
toll-like receptor TLR1:TLR2 signaling pathway (GO:0038123)
epidermal growth factor receptor signaling pathway (GO:0007173)
positive regulation of neuron projection development (GO:0010976)
TRIF-dependent toll-like receptor signaling pathway (GO:0035666)
stimulatory C-type lectin receptor signaling pathway (GO:0002223)
C1
C2
C3
C4
SE(C1)
SE(C2)
SE(C3)
SE(C4)
SN(C1)
SN(C2)
SN(C3)
SN(C4)
