# Supplementary material for: Functional networks inference from rule-based machine learning models
Source: BioData Min. 2016 Sep 5;9:28. doi: 10.1186/s13040-016-0106-4 (PMC5011349; doi:10.1186/s13040-016-0106-4)
Supplement: Supplementary file 4 — Enriched terms. Complete list of enriched terms for each dataset and network. (XZ 3041 kb) [file 13040_2016_106_MOESM4_ESM.xz › enriched_terms/co_prediction/heatmap_mic_p.pdf]

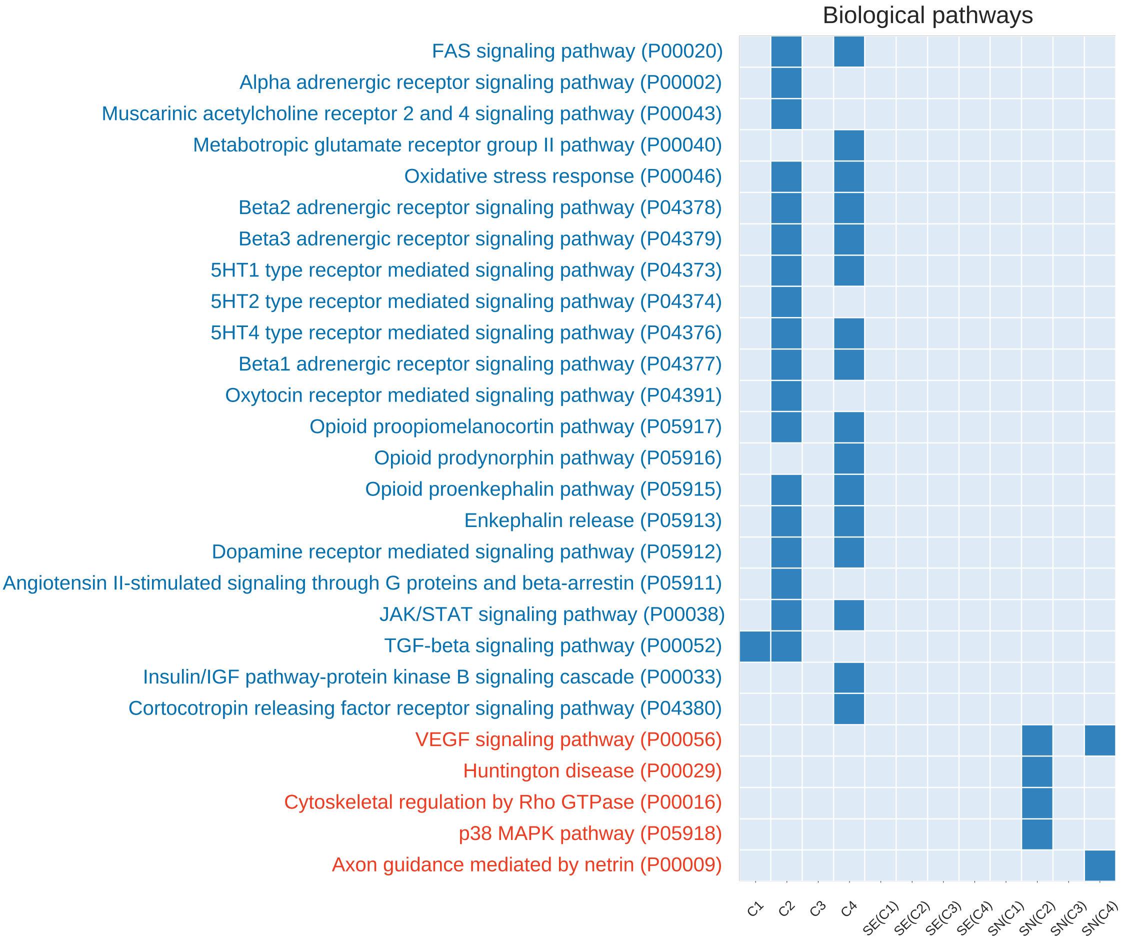
Biological pathways
FAS signaling pathway (P00020)
Alpha adrenergic receptor signaling pathway (P00002)
Muscarinic acetylcholine receptor 2 and 4 signaling pathway (P00043)
Metabotropic glutamate receptor group II pathway (P00040)
Oxidative stress response (P00046)
Beta2 adrenergic receptor signaling pathway (P04378)
Beta3 adrenergic receptor signaling pathway (P04379)
5HT1 type receptor mediated signaling pathway (P04373)
5HT2 type receptor mediated signaling pathway (P04374)
5HT4 type receptor mediated signaling pathway (P04376)
Beta1 adrenergic receptor signaling pathway (P04377)
Oxytocin receptor mediated signaling pathway (P04391)
Opioid proopiomelanocortin pathway (P05917)
Opioid prodynorphin pathway (P05916)
Opioid proenkephalin pathway (P05915)
Enkephalin release (P05913)
Dopamine receptor mediated signaling pathway (P05912)
Angiotensin II-stimulated signaling through G proteins and beta-arrestin (P05911)
JAK/STAT signaling pathway (P00038)
TGF-beta signaling pathway (P00052)
Insulin/IGF pathway-protein kinase B signaling cascade (P00033)
Cortocotropin releasing factor receptor signaling pathway (P04380)
VEGF signaling pathway (P00056)
Huntington disease (P00029)
Cytoskeletal regulation by Rho GTPase (P00016)
p38 MAPK pathway (P05918)
Axon guidance mediated by netrin (P00009)
C1
C2
C3
C4
SE(C1)
SE(C2)
SE(C3)
SE(C4)
SN(C1)
SN(C2)
SN(C3)
SN(C4)
