# Supplementary material for: Functional networks inference from rule-based machine learning models
Source: BioData Min. 2016 Sep 5;9:28. doi: 10.1186/s13040-016-0106-4 (PMC5011349; doi:10.1186/s13040-016-0106-4)
Supplement: Supplementary file 4 — Enriched terms. Complete list of enriched terms for each dataset and network. (XZ 3041 kb) [file 13040_2016_106_MOESM4_ESM.xz › enriched_terms/co_prediction/heatmap_mic_bp.pdf]

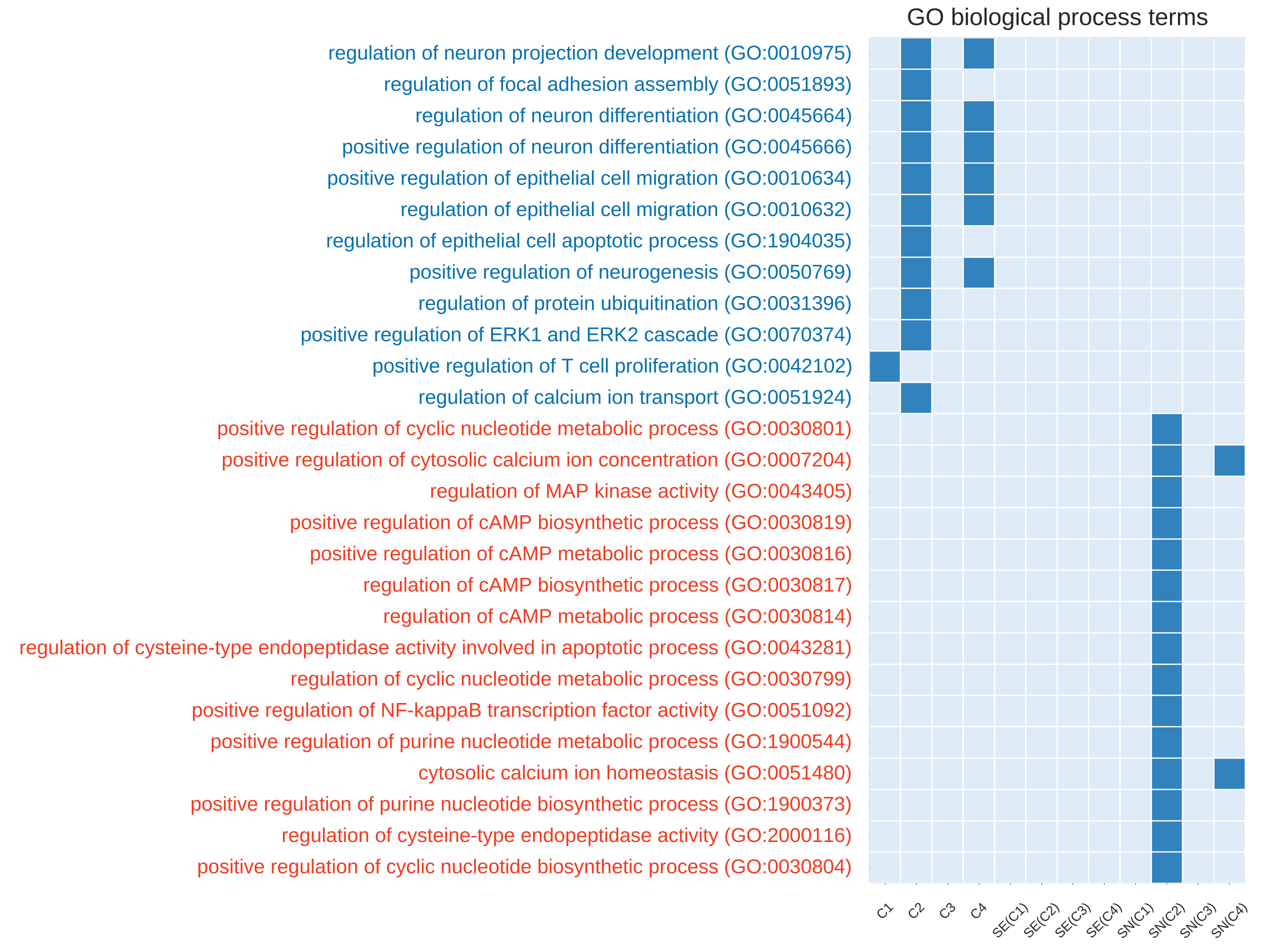
GO biological process terms
regulation of neuron projection development (GO:0010975)
regulation of focal adhesion assembly (GO:0051893)
regulation of neuron differentiation (GO:0045664)
positive regulation of neuron differentiation (GO:0045666)
positive regulation of epithelial cell migration (GO:0010634)
regulation of epithelial cell migration (GO:0010632)
regulation of epithelial cell apoptotic process (GO:1904035)
positive regulation of neurogenesis (GO:0050769)
regulation of protein ubiquitination (GO:0031396)
positive regulation of ERK1 and ERK2 cascade (GO:0070374)
positive regulation of T cell proliferation (GO:0042102)
regulation of calcium ion transport (GO:0051924)
positive regulation of cyclic nucleotide metabolic process (GO:0030801)
positive regulation of cytosolic calcium ion concentration (GO:0007204)
regulation of MAP kinase activity (GO:0043405)
positive regulation of cAMP biosynthetic process (GO:0030819)
positive regulation of cAMP metabolic process (GO:0030816)
regulation of cAMP biosynthetic process (GO:0030817)
regulation of cAMP metabolic process (GO:0030814)
regulation of cysteine-type endopeptidase activity involved in apoptotic process (GO:0043281)
regulation of cyclic nucleotide metabolic process (GO:0030799)
positive regulation of NF-kappaB transcription factor activity (GO:0051092)
positive regulation of purine nucleotide metabolic process (GO:1900544)
cytosolic calcium ion homeostasis (GO:0051480)
positive regulation of purine nucleotide biosynthetic process (GO:1900373)
regulation of cysteine-type endopeptidase activity (GO:2000116)
positive regulation of cyclic nucleotide biosynthetic process (GO:0030804)
C1
C2
C3
C4
SE(C1)
SE(C2)
SE(C3)
SE(C4)
SN(C1)
SN(C2)
SN(C3)
SN(C4)
