# Supplementary material for: Functional networks inference from rule-based machine learning models
Source: BioData Min. 2016 Sep 5;9:28. doi: 10.1186/s13040-016-0106-4 (PMC5011349; doi:10.1186/s13040-016-0106-4)
Supplement: Supplementary file 4 — Enriched terms. Complete list of enriched terms for each dataset and network. (XZ 3041 kb) [file 13040_2016_106_MOESM4_ESM.xz › enriched_terms/co_prediction/rank_malacards.pdf]

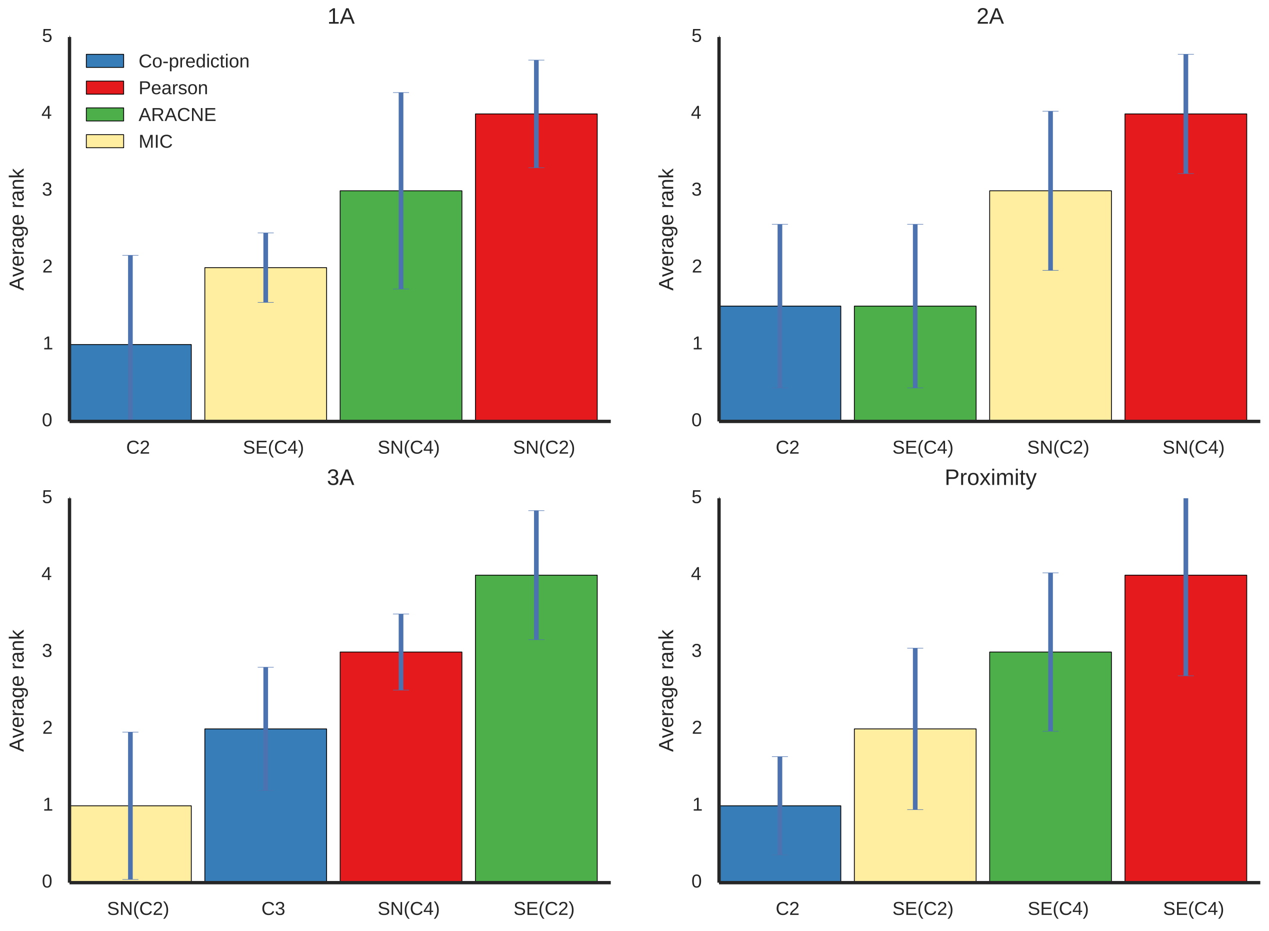
1A
2A
3A
Proximity
Average rank
0
1
2
3
4
5
Co-prediction
Pearson
ARACNE
MIC
C2
SE(C4)
SN(C4)
SN(C2)
C2
SE(C4)
SN(C2)
SN(C4)
SN(C2)
C3
SN(C4)
SE(C2)
C2
SE(C2)
SE(C4)
SE(C4)
